# Identification of Cleavage Sites Recognized by the 3C-Like Cysteine Protease within the Two Polyproteins of Strawberry Mottle Virus

**DOI:** 10.3389/fmicb.2017.00745

**Published:** 2017-04-27

**Authors:** Krin S. Mann, Melanie Walker, Hélène Sanfaçon

**Affiliations:** Agriculture and Agri-Food Canada, Summerland Research and Development Centre, SummerlandBC, Canada

**Keywords:** proteolytic processing, viral proteases, plant virus, cleavage site specificity, 3C protease, picornavirales, secoviridae, *in vitro* translation

## Abstract

Strawberry mottle virus (SMoV, family *Secoviridae*, order *Picornavirales*) is one of several viruses found in association with strawberry decline disease in Eastern Canada. The SMoV genome consists of two positive-sense single-stranded RNAs, each encoding one large polyprotein. The RNA1 polyprotein (P1) includes the domains for a putative helicase, a VPg, a 3C-like cysteine protease and an RNA-dependent RNA polymerase at its C-terminus, and one or two protein domains at its N-terminus. The RNA2 polyprotein (P2) is predicted to contain the domains for a movement protein (MP) and one or several coat proteins at its N-terminus, and one or more additional domains for proteins of unknown function at its C-terminus. The RNA1-encoded 3C-like protease is presumed to cleave the two polyproteins *in cis* (P1) and *in trans* (P2). Using *in vitro* processing assays, we systematically scanned the two polyproteins for cleavage sites recognized by this protease. We identified five *cis*-cleavage sites in P1, with cleavage between the putative helicase and VPg domains being the most efficient. The presence of six protein domains in the SMoV P1, including two upstream of the putative helicase domain, is a feature shared with nepoviruses but not with comoviruses. Results from *trans*-cleavage assays indicate that the RNA1-encoded 3C-like protease recognized a single cleavage site, which was between the predicted MP and coat protein domains in the P2 polyprotein. The cleavage site consensus sequence for the SMoV 3C-like protease is AxE (E or Q)/(G or S).

## Introduction

Strawberry decline disease has emerged as a significant problem for strawberry production in Eastern Canada and is likely caused by the synergistic effects of mixed virus infections. Strawberry mottle virus (SMoV) is one of the viruses found in association with this disease (Martin and Tzanetakis, 2013). The species *Strawberry mottle virus* has been classified within the family *Secoviridae* (order *Picornavirales*) but is currently not assigned to a specific genera, mostly because its genomic organization has not yet been clarified (Sanfacon et al., 2011; Sanfacon, 2015). Similar to the majority of members of the family *Secoviridae* (referred to as secovirids), the SMoV genome consists of two positive sense RNA molecules. Each RNA encodes one large polyprotein referred to as P1 (∼215 kDa) and P2 (∼190 kDa) for RNA1 and RNA2, respectively (Thompson et al., 2002; Sanfacon, 2015; Bhagwat et al., 2016). The two polyproteins are presumably cleaved by an RNA1-encoded 3C-like protease (related to the 3C proteases of picornaviruses) (Gorbalenya et al., 1989) to release mature proteins and intermediate precursor proteins made up of two or more protein domains. The active site of the 3C and 3C-like proteases is typified by having a cysteine residue and is structurally related to the trypsin-like family of serine proteases (Bazan and Fletterick, 1988; Dougherty et al., 1989; Baum et al., 1991). Common dipeptides recognized by 3C or 3C-like proteases include Q/G, Q/S, and E/G (Wellink and van Kammen, 1988; Gorbalenya et al., 1989; Seipelt et al., 1999; Sanfacon et al., 2011; Sanfacon, 2015). The specificity for a glutamine (Q) or glutamate (E) at the -1 position of the cleavage site is conferred by the conserved histidine in the substrate-binding pocket of the protease, which is also present in the SMoV protease (Bazan and Fletterick, 1988; Allaire et al., 1994; Sanfacon et al., 2011).

A common feature of secovirids is that the C-terminal region of the P1 polyprotein includes functional domains for a type III putative RNA helicase (also termed NTB, for NTP-binding protein), a viral genome-linked protein (VPg), a 3C-like protease (Pro), and a type I RNA-dependent RNA polymerase (Pol) (Sanfacon et al., 2009, 2011). The region upstream of the NTB domain is more variable and less well conserved for secovirids. The N-terminal region of comovirus polyproteins contains a single protein domain termed Co-Pro or 32K protein, which is involved in regulating the protease activity (Peters et al., 1992). In contrast, nepoviruses possess two protein domains (i.e., X1 and X2) upstream of NTB (Wang and Sanfacon, 2000; Wetzel et al., 2008). The nepovirus X2 domain has sequence homologies with the comovirus Co-Pro, although there is no evidence that it regulates the activity of Pro. Similar to the comovirus Co-Pro, the nepovirus X2 protein is an endoplasmic reticulum-associated integral membrane protein that likely plays a role in virus replication (Carette et al., 2002; Zhang and Sanfacon, 2006; Sanfacon, 2013). The biological function of the nepovirus X1 domain is not known. Based on sequence alignments, putative Q/G cleavage sites have been proposed in the SMoV P1 polyprotein that would define the NTB, VPg, Pro, and Pol domains and a putative Co-Pro domain upstream of NTB (Thompson et al., 2002). However, these have not been confirmed experimentally and the possibility that additional protein domains exist upstream of the NTB domain has not been investigated.

Similar to other secovirids, the SMoV P2 polyprotein contains the domains for a movement protein (MP) and capsid protein (CP) (Sanfacon et al., 2009; Bhagwat et al., 2016). Secovirids encode either one large 55–60 kDa CP (nepoviruses), two with molecular masses of approximately 40 and 20 kDa (comoviruses, fabaviruses, sadwaviruses, and strawberry latent ringspot virus) or three small 20–25 kDa CPs (cheraviruses, torradoviruses, sequiviruses, and waikaviruses) (Sanfacon et al., 2009, 2011). However, the number of CP(s) encoded by SMoV and the related black raspberry necrosis virus (BRNV) is not known. Efforts to clarify the number of CPs for these viruses have been hampered by the low virus titers in infected plants which preclude purification of virus particles (Thompson et al., 2002; Halgren et al., 2007). For all characterized bipartite secovirids, the MP and CP domains are present in the C-terminal region of P2, with a variable number of protein domains upstream of the MP domain (Sanfacon et al., 2011). However, a region having sequence identity to the two coat protein domains of satsuma dwarf virus (SDV, a related sadwavirus) was found in the central region of the SMoV RNA2 polyprotein rather than at the C-terminus of the polyprotein (Karasev et al., 2001; Thompson et al., 2002; Bhagwat et al., 2016). Interestingly, the C-terminal region of the P2 polyprotein of five Canadian isolates of SMoV is even larger than that previously observed for isolate 1134 from the Netherlands (Bhagwat et al., 2016), with a total coding capacity of approximately 70 kDa downstream of the presumed CP domain. The biological function of the C-terminal domain of P2 is not known. An E/G cleavage site was proposed between the MP and CP domains (Thompson et al., 2002). Additional cleavage sites located within the putative CP domain (to delineate two or more possible CPs) or downstream of this domain were not confidently predicted (Thompson et al., 2002; Bhagwat et al., 2016).

In this study, we sought to characterize the proteolytic processing of SMoV polyproteins and define functional protein domains. Using *in vitro* processing assays, we identified five *cis*-cleavage sites that are recognized by the 3C-like protease in the P1 polyprotein, delineating six protein domains including two upstream of the NTB domain. *Trans*-cleavage at the predicted E/G site between the MP and CP domains was confirmed, but no other *trans-*cleavage sites were found to be recognized by the RNA1-encoded 3C-like protease in the P2 polyprotein.

## Materials and Methods

### Cloning of SMoV Partial Polyprotein Precursor Constructs

The complete genomic sequence of SMoV Nova Scotia isolate NSPer3 (accession numbers, KU200456-KU200457">KU200456-KU200457) has been described previously (Bhagwat et al., 2016) and this isolate was the source for all constructs described below. Reverse transcription was conducted using SuperScript IV (Thermo Fisher) and primer P610R (see **Table 1** for primers) to generate cDNA which was then used as a template for PCR amplification using Q5 HF DNA polymerase (New England Biolabs). RT-PCR fragments corresponding to constructs NTB’-Pol’, 365-735, 1-865 and 501-1691 were generated using specific primers that included additional restriction sites for cloning into vector pCITE4a (Novagen) (**Table 1**). Fragments for all other constructs were synthesized commercially by GeneArt (Thermo Fisher) and were subsequently subcloned into pCITE4a. Fragments were inserted into the *Bam*HI-*Xho*I (NTB’-Pol’, 365-735, 1-865, and 501-1691) or the *Nco*I-*Bam*HI restriction sites (most other constructs) of the pCITE4a polylinker, resulting in an N-terminal in frame fusion to the S-tag contained within the vector. The only exception was the VPg-Pro construct, which was inserted into the *Msc*I-*Bam*HI sites of pCITE4a, allowing the synthesis of a viral protein with only three additional amino acids at its N-terminus (including one additional methionine as a start codon). Therefore, this construct (and mutated derivative) was not fused to the S-tag so as to facilitate expression of the native protease. In all cases, a stop codon was inserted immediately downstream of the viral sequence. Mutations were inserted into the parent constructs either by site-directed mutagenesis (Fisher and Pei, 1997) using specific primers or using the mutagenesis service of GeneArt. DNA sequence for all constructs was verified by Sanger Sequencing using the ABI 3500 series Genetic Analyzer (Thermo Fisher).

**Table 1:** List of primers used in this study.

Name	Sequence	Construct
P610R	CATATTTACATCTATCTAAAGTTAAG	SMoV cDNA synthesis
P534F	CTAGTAGGATCCATGTCAAAGGTTATGGCCATGCACAC	NTB’-Pol’
P548R	CGCTCGAGTTAAATTTTGACGACCATTT	
P645F	CTAGTAGGATCCCCAATACACCCATACCGGGCAA	365–735
P646R	CGCTCGAGaaaTCATTGACCTGCAACACTACTCCTG	
P613R-PER3-R2	CGCTCGAGTTAGATAACACGTTCCACCCA	1–865
P614F-PER3-R2	CTAGTAGGATCCATGTTTTTATTTGTGTTGTATGCTTGCC	
P620F-PER3	CTAGTAGGATCCATGGAAGACACCGACGTTGCCAATT	501–1691
P621-R-PER3	CGCTCGAGTCACAACCCTAAGGCCCCA	

*Restriction enzyme sites (red) and start/stop codons (purple) are highlighted.*

### Multiple Sequence Alignments

The deduced amino acid sequence of the P1 and P2 polyproteins from SMoV NSPer3, BRNV isolate Alyth (accession numbers, FN908128-FN908129">FN908128-FN908129), tomato ringspot virus isolate Rasp2 (ToRSV-Rasp2, NC_003839-NC_003840), arabis mosaic virus isolate NW (ArMV-NW, NC_006056-NC_006057), chocolate lily virus A (CLVA, NC_016443-NC_016444) and dioscorea mosaic-associated virus (DMaV, KU215538-KU215539) were used for **Table 2** and for the alignments shown in the Supplementary Figures S1, S2. Multiple amino acid sequence alignments were produced using Clustal Omega (Sievers et al., 2011).

**Table 2:** Alignment of potential cleavage sites for members of the family *Secoviridae.*

	SMoV	BRNV	CLVA	DMaV
**P1 sites**				
X1-X2	AQCVEQ/GG	IGLEQE/GF	EHETCQ/GL	SEEELQ/GL
X2-NTB	CPAYEQ/SS	DVPLSE/GA	ADVAAQ/SG	EPMMLQ/AG
NTB-VPg	EVATEQ/GG	LAFTSE/GG	SSSLAQ/GT	RGFQLQ/GG
VPg-Pro	VRAYEQ/GA	IKPYSQ/GG	RAFSAQ/GE	RGFQLQ/GG
Pro-Pol	EVAVQQ/GM	GKFYQQ/GD	PVIVAQ/GP	PSDDLQ/SE
**P2 sites**				
MP-CP	TRAYEE/GL	DDFVEE/GD	GDAAAQ/GD	LNDSLE/GD

*Amino acids highlighted in red represents highly conserved positions. Amino acids highlighted in green represent alternative amino acids found at the -4, -2, -1, or +1 positions. Strawberry mottle virus (SMoV); black raspberry necrosis virus (BRNV); chocolate lily virus A (CLVA); dioscorea mosaic-associated virus (DMaV); NTB, nucleoside triphosphate binding protein; VPg, viral genome-linked protein; Pro, protease; Pol, polymerase; X1 and X2, unknown; MP, movement protein; CP, capsid protein.*

### *In Vitro* Translation Reactions

For *in vitro* translation assays, the rabbit reticulocyte system was chosen because wheat germ extracts were previously reported to contain inhibitors of 3C-like proteases (Shih et al., 1987; Margis et al., 1991). Protein translation reactions were carried out using the TnT Quick coupled transcription/translation rabbit reticulocyte system (Promega) as previously described (Wetzel et al., 2013). Briefly, protein labeling with EasyTag L-[^35^S]-methionine (PerkinElmer) was carried out at 29°C for 90 min followed by translation termination by the addition of an RNase A and cold methionine mix. *Cis*-cleavage reactions were directly diluted in an equal volume of protease buffer [10 mM HEPES, pH 6.5, 0.1% CHAPS (3-[(3-cholamidopropyl)dimethylammonio]-1-propanesulfonate hydrate), 10 mM DTT (dithiothreitol) and 30% glycerol] (May et al., 2014) and incubated at 16°C overnight to facilitate proteolytic processing. For *trans*-cleavage reactions, samples were prepared by mixing the unlabelled VPg-Pro translation product with [^35^S]-methionine labeled products from RNA2 construct(s) at a ratio of 5:1. The mixture was then diluted in an equal volume of protease buffer and incubated at 16°C overnight. Following overnight incubation, an equal volume of 2X SDS protein loading buffer was added (Laemmli, 1970). Samples were heated at 60°C for 10 min followed by separation by 10 or 12% SDS-polyacrylamide gel electrophoresis (SDS-PAGE). Protein bands were visualized using a phosphoimager (Cyclone Plus, PerkinElmer).

## Results

### Definition of the VPg, Pro, and Pol Domains Using a Truncated Precursor Polyprotein

**FIGURE 1:**
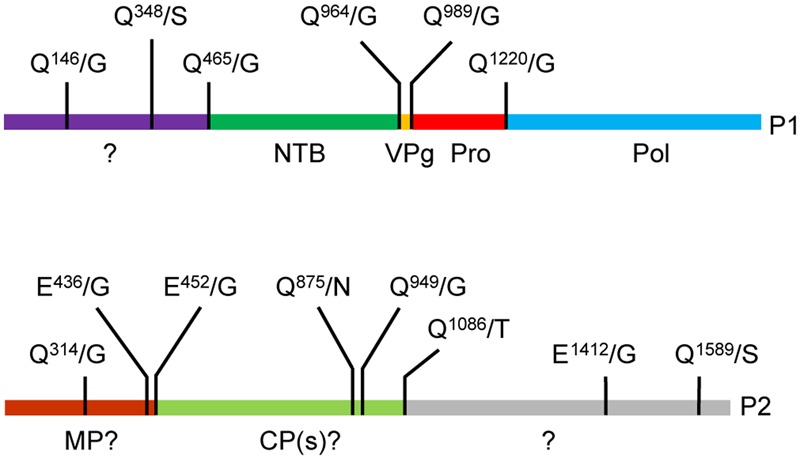
**Predicted cleavage sites in the strawberry mottle virus (SMoV) NSPer3 P1 and P2 polyproteins.** Predicted cleavage sites are shown as short vertical lines above the full-length polyproteins along with the predicted cleaved dipeptide. Numbering of amino acids are defined from the beginning of each polyprotein. Please note that the numbering is identical for all SMoV isolates, although the P2 polyprotein of Netherlands isolate 1134 does not include the Q^1589^/S sequence due to a truncation of its C-terminal region. Deduced functional domains based on sequence homologies with related viruses are listed as follows. NTB, nucleoside triphosphate binding protein or putative helicase; VPg, viral genome-linked protein; Pro, protease; Pol, polymerase; MP, movement protein; CP, capsid protein.

Based on alignments of P1 polyproteins amongst Canadian SMoV isolates and with related secovirids, putative cleavage sites were previously predicted including Q^465^/G, Q^964^/G, Q^989^/G, and Q^1220^/G (**Figure 1**, numbering correspond to the amino acid position starting from the beginning of the polyprotein) (Thompson et al., 2002; Bhagwat et al., 2016). The last three proposed cleavage sites would delineate the VPg, Pro, and Pol domains, while the first cleavage site was tentatively proposed to be upstream of the NTB domain. Re-examination of amino acid sequence alignments revealed that two additional possible cleavage sites were present: Q^146^/G and Q^348^/S, which correspond approximately to the positions of the previously characterized X1-X2 and X2-NTB cleavage sites of two nepoviruses [ToRSV and ArMV] (Wang and Sanfacon, 2000; Wetzel et al., 2008) (Supplementary Figure S1).

**FIGURE 2:**
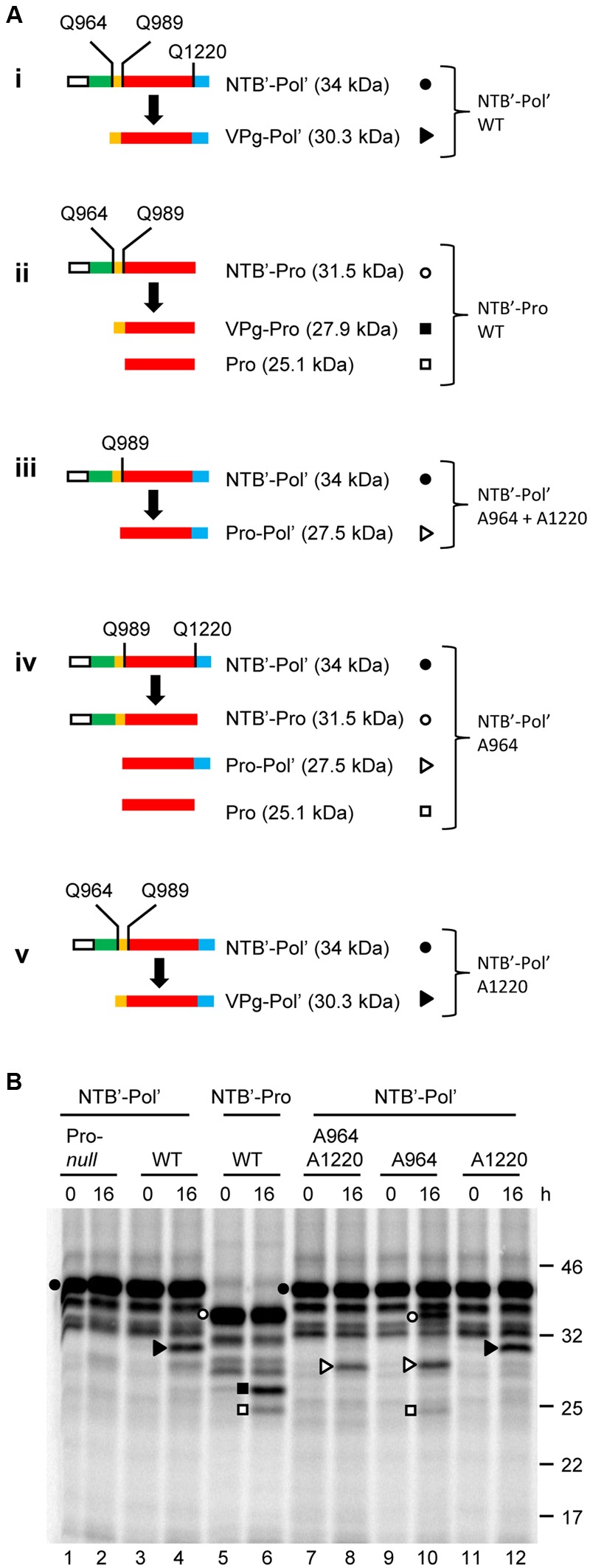
***In vitro cis*-processing of the partial P1 polyprotein precursor clone NTB’-Pol’ delineates the VPg, Pro, and Pol domains. (A)** A schematic representation of the wild-type or mutated derivatives of the NTB’-Pol’ (i and iii–v) or NTB’-Pro (ii) precursor polyproteins and the detected cleavage products is shown. Vertical lines represent cleavage sites with the amino acid at the –1 position indicated for each site (numbering of amino acids based on their position in the P1 polyprotein). In the case of cleavage site mutants, only the remaining wild-type cleavage sites are shown. Deduced functional domains are shown as follows: NTB (green), VPg (yellow), Pro (red), and Pol (blue). The white box represents the S-tag which was fused in frame at the N-terminus of the polyprotein. **(B)** *In vitro* processing reactions of the wild-type or mutant precursor polyprotein clone NTB’-Pol’ were incubated overnight at 16°C followed by separation of the precursor polyprotein and potential cleavage products by 12% SDS-PAGE. Cleavage products are represented by symbols on the right of each lane as shown in the schematic representation **(A)**. The migration positions of molecular mass markers (kDa) are indicated on the right of the gel.

Proteolytic processing of SMoV P1 was initially investigated using a partial polyprotein precursor containing the entire VPg and Pro domains as well as a portion of the C-terminal region of the NTB domain (NTB’) and N-terminal region of the Pol domain (Pol’). This construct, referred to as NTB’-Pol’, included the predicted NTB-VPg, VPg-Pro and Pro-Pol cleavage sites (**Figure 2A**, i). To confirm that any detected proteolytic cleavage was due to the activity of the 3C-like Pro, we also generated a mutant derivative of the NTB’-Pol’ construct in which the conserved cysteine of the catalytic triad (C1171) was mutated to alanine (referred to as Pro-*null*). Results from the *in vitro* translation assays showed accumulation of the expected precursor polyprotein (34 kDa) after a 90 min translation reaction (**Figure 2B**, 0 h, lane 1). A few smaller minor bands were also observed but were likely not due to a specific proteolytic event directed by the 3C-like protease, since they were also observed in the Pro*-null* derivative (**Figure 2B**, compare lanes 1 and 3). Rather, they were likely the result of internal translation initiation or premature translation termination events. After an overnight incubation in the protease buffer (see Materials and Methods), a predominant cleavage product was observed for the wild-type NTB’-Pol’ but not for the Pro-*null* derivative (**Figure 2B**, 16 h, compare lanes 2 and 4). This band had an apparent molecular mass of 30 kDa, which is close to the calculated size for the predicted VPg-Pol’ cleavage product (30.3 kDa) after processing at the NTB-VPg cleavage site (**Figure 2B**, lane 4). Other possible cleavage products that could arise from secondary cleavage at the remaining VPg-Pro and/or Pro-Pol cleavage sites (Pro-Pol’, VPg-Pro, and Pro) were not confidently detected over the background.

Next, we tested a truncated precursor (NTB’-Pro) which lacks the Pol’ sequence, and therefore the Pro-Pol cleavage site (**Figure 2A**, ii). As above, primary cleavage at the predicted NTB-VPg site was detected resulting in the accumulation of a protein corresponding in size to the VPg-Pro cleavage product after an overnight incubation (**Figure 2B**, lane 6). In addition, smaller amounts of another cleavage product were detected that likely corresponded to the mature Pro (25.1 kDa) after secondary processing at the VPg-Pro cleavage site. The other expected cleavage product, i.e., the mature VPg protein, was not detected from these gels due to its small size (2.8 kDa).

We further characterized the processing of NTB’-Pol’ by introducing mutations in potential cleavage sites. We chose to mutate the conserved glutamine (Q) of the -1 position of the cleavage site to an alanine (**Figure 2A**, iii–v). A similar mutation in ToRSV cleavage sites was previously shown to completely abolish *cis-* or *trans-*cleavage (Carrier et al., 1999). Single mutation of the predicted Pro-Pol cleavage site (A1220 mutant, **Figure 2A**, v) did not drastically change the cleavage pattern compared to the wild-type polyprotein and resulted in accumulation of the VPg-Pol’ product after cleavage at the predominant NTB-VPg site (**Figure 2B**, compare lanes 4 and 12). As expected, single mutation of the predicted NTB-VPg cleavage site (A964 mutant, **Figure 2A**, iv) prevented the accumulation of the VPg-Pol’ cleavage product (**Figure 2B**, compare lanes 4 and 10). Instead, the mutation resulted in cleavage at the remaining VPg-Pro and Pro-Pol sites (**Figure 2A**, iv). This was evidenced by the accumulation of cleavage products corresponding in size to NTB’-Pro (31.5 kDa), Pro-Pol’ (27.5 kDa) and Pro (25.1 kDa) (**Figure 2B**, lane 10). Mutation of both the NTB-VPg and Pro-Pol cleavage sites (A964 + A1220 double mutant, **Figure 2A**, iii) resulted in cleavage at the only available cleavage site between VPg and Pro resulting in the accumulation of the Pro-Pol’ cleavage product (27.5 kDa) (**Figure 2B**, lane 8). The presence of a large amount of the precursor polyprotein after an overnight incubation for all tested wild-type or mutated NTB’-Pol’ derivatives (**Figure 2B**, lanes 2, 4, 6, 8, 10, and 12) suggests that *in vitro* cleavage by the 3C-like Pro is not efficient. Despite this observation, our results confirm cleavage of the NTB’-Pol’ construct by the 3C-like Pro at the three predicted cleavage sites, with the NTB-VPg cleavage site being recognized the most efficiently at least *in vitro*.

### Identification of Two Additional Cleavage Sites Upstream of the NTB Domain

**FIGURE 3:**
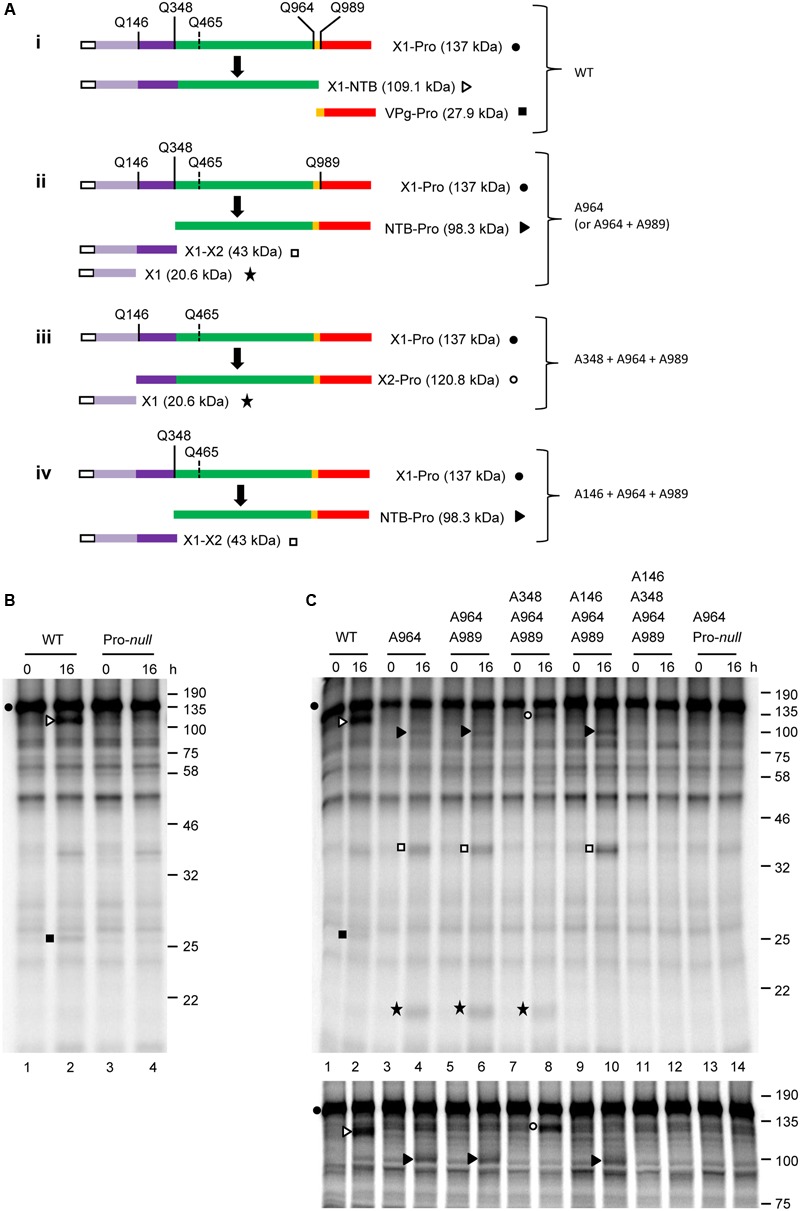
***In vitro cis*-processing of the X1-Pro polyprotein precursor reveals two additional cleavage sites upstream of the NTB domain. (A)** A schematic representation of the X1-Pro precursor polyproteins (wild-type, i and mutant derivatives, ii–iv) and the detected cleavage products is shown. Cleavage sites are depicted as described in **Figure 2**. Please note that the predicted Q^465^/G cleavage site is shown with a dashed line since processing was not detected at this site. Deduced functional protein domains are shown as follows: X1 (light purple), X2 (dark purple), NTB (green), VPg (yellow), and Pro (red). The white box represents the S-tag which was fused in frame at the N-terminus of the polyprotein. **(B)** *In vitro* processing reactions of the wild-type or Pro-*null* X1-Pro polyprotein clones were incubated overnight at 16°C followed by separation of the precursor polyprotein and potential cleavage products by 12% SDS-PAGE. **(C)** *In vitro* processing reactions of the wild-type or mutant X1-Pro precursors were incubated overnight at 16°C, followed by separation of the precursor polyprotein and potential cleavage products by 12% SDS-PAGE (upper panel) or 10% SDS-PAGE (lower panel). **(B,C)** Cleavage products are represented by symbols on the right side of each lane as shown in the schematic representation **(A)**. The migration positions of molecular mass markers (kDa) are indicated on the right of the gels.

To determine whether proteolytic processing can occur at other predicted P1 polyprotein cleavage sites, we generated a construct that spans the entire N-terminal region of the P1 polyprotein up to and including the Pro domain (X1-Pro; 137 kDa) (**Figure 3A**, i). Although the X1-Pro precursor included five possible cleavage sites, the only detectable cleavage observed was between NTB and VPg. This was determined based on the accumulation of cleavage products that corresponded in size to X1-NTB (109.1 kDa) and VPg-Pro (27.9 kDa) (**Figure 3B**, lane 2). These cleavage products were not detected in the Pro-*null* derivative (**Figure 3B**, lane 4), confirming that cleavage was due to the activity of the 3C-like Pro. Taken together, these results are similar to those observed with the NTB’-Pol’ construct and indicate that the SMoV 3C-like Pro preferentially cleaves at the NTB-VPg site *in vitro*.

Since suboptimal cleavage events were detectable when one or more cleavage sequences were mutated in the NTB’-Pol’ construct (**Figure 2**), we used a similar strategy to complete the mapping of the N-terminal domain(s) of P1. Using the X1-Pro construct, a series of constructs with mutations at one or more cleavage sites were generated (**Figure 3A**, ii–iv). For each mutant tested, translation reactions were ran simultaneously on a 12% SDS-acrylamide gel to visualize smaller cleavage products (**Figure 3C**, upper panel) and on a 10% gel to optimize the separation of larger cleavage products (**Figure 3C**, lower panel). Compared to the wild-type, mutation of the NTB-VPg cleavage sequence (A964 mutant, **Figure 3A**, ii) prevented the release of the X1-NTB and VPg-Pro cleavage products (**Figure 3C**, compare lanes 2 and 4). Instead, new cleavage products were observed. A band with an apparent molecular mass of approximately 100 kDa likely corresponds to NTB-Pro, suggesting that cleavage upstream of the NTB domain occurred at Q^348^/S (calculated molecular mass for NTB-Pro of 98.3 kDa) rather than at the originally predicted Q^465^/G (calculated molecular mass for NTB-Pro of 84.8 kDa). Two other major cleavage products were also detected. The first cleavage product migrated at approximately 38 kDa and could correspond to the entire N-terminal region of P1 upstream of Q^348^/S (calculated molecular mass of 43 kDa). The second one was approximately 20 kDa and could result from cleavage at Q^146^/G, which would define two small protein domains upstream of NTB. We will refer to these domains as X1 (20.6 kDa) and X2 (22.3 kDa), by analogy to the X1 and X2 domains mapped in the N-terminal region of the P1 polyproteins of two nepoviruses (Wang and Sanfacon, 2000; Wetzel et al., 2008). Introduction of the Pro*-null* mutation in the X1-Pro A964 mutant (double mutant A964 + Pro-*null*) prevented the accumulation of these new cleavage products, confirming that the activity of the 3C-like Pro is required (**Figure 3C**, lane 14).

In contrast to results with the smaller NTB’-Pol’ precursor (**Figure 2**), cleavage was not observed between the VPg and Pro domains in the X1-Pro polyprotein after mutation of the NTB-VPg site, as release of the mature Pro was not detected for the A964 mutant (**Figure 3C**, lane 4). This suggests that the VPg-Pro cleavage site may be suboptimal in the presence of the X1-X2 and X2-NTB cleavage sites. Indeed, introducing a second mutation in the VPg-Pro cleavage site in addition to the NTB-VPg cleavage site mutation (double mutant A964 + A989) did not alter the cleavage product banding pattern (**Figure 3C**, compare lanes 4 and 6). To confirm that cleavage occurred between X2 and NTB at Q^348^/S, and between X1 and X2 at Q^146^G, we introduced mutations of these cleavage sites in the X1-Pro double mutant (A964 + A989), creating triple and quadruple mutants. Mutation of the X2-NTB Q^348^/S cleavage site (triple mutant A348 + A964 + A989, **Figure 3A**, iii) resulted in the loss of the X1-X2 cleavage product and processing at the cleavage site between X1 and X2. This was evidenced by the accumulation of two cleavage products corresponding to X2-Pro (120.8 kDa) and X1 (**Figure 3C**, lane 8). Similarly, mutation of the X1-X2 Q^146^/G cleavage site (triple mutant A146 + A964 + A989, **Figure 3A**, iv) resulted in the loss of the X1 product and processing at the X2-NTB cleavage site (Q^348^/S), resulting in the accumulation of NTB-Pro and X1-X2 (**Figure 3C**, lane 10). A quadruple mutant with simultaneous mutation of the Q^146^/G, Q^348^/S, Q^964^/G, and Q^989^/G cleavage sites was not cleaved by the 3C-like protease (**Figure 3C**, lane 12) suggesting that the putative Q^465^/G site was not recognized. Taken together, our results identify a total of five cleavage sites in the P1 polyprotein with the consensus sequence AxEQ/(G or S) (**Table 2**). Similar to what was observed for two nepoviruses, these cleavage sites define six protein domains in the P1 polyprotein, namely X1, X2, NTB, VPg, Pro, and Pol.

### SMoV RNA2 Polyprotein Is Cleaved between the MP and CP Domains by the RNA1-Encoded 3C-Like Protease

**FIGURE 4:**
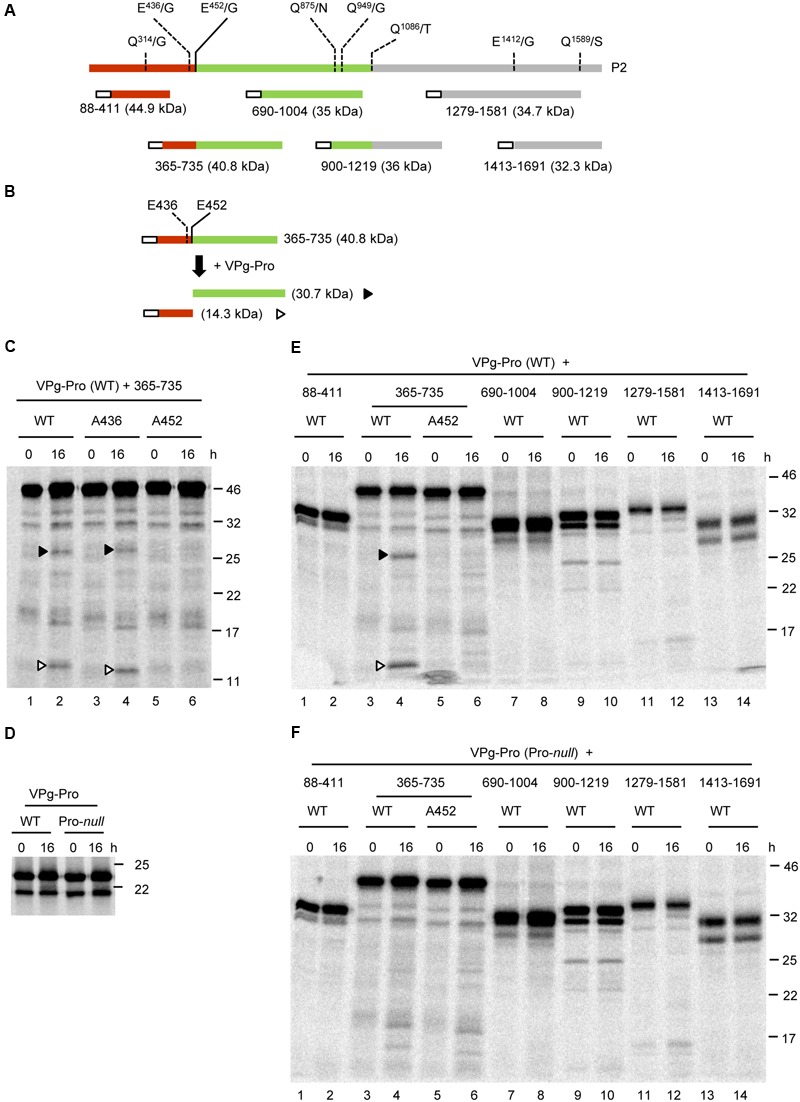
***In vitro trans*-processing assays of partial P2 polyprotein precursor clones identify a single cleavage event between the MP and CP domains. (A)** Schematic representation of the P2 polyprotein and the collection of overlapping partial polyprotein precursors. The predicted MP and CP domains are shown in brown and green, respectively. Putative cleavage sites are shown with the vertical lines and dashed lines represent cleavage sites that were not processed in the *in vitro* assays. The white box represents the S-tag which was fused in frame at the N-terminus of the polyproteins. **(B)** Schematic representation of the 365-735 precursor and the detected cleavage products is shown. Predicted cleavage sites are shown as described in **Figure 2**. **(C)** *In vitro* processing reactions of the wild-type or mutant 365-735 precursor in the presence of unlabelled VPg-Pro were incubated overnight at 16°C, followed by separation of the polyprotein precursor and potential cleavage products by 12% SDS-PAGE. **(D)** *In vitro* translation of the VPg-Pro construct in presence of [^35^S]-methionine confirmed similar expression levels of the wild-type or Pro-*null* derivatives. **(E,F)** *In vitro* processing of the collection of partial P2 polyprotein precursors were incubated overnight in the presence of the wild-type VPg-Pro **(E)** or the Pro-*null* derivative of VPg-Pro **(F). (C–E)** Cleavage products are represented by symbols on the right side of each lane as shown in the schematic representation **(B)**. The migration positions of molecular mass markers (kDa) are indicated in the right of the gels.

Scanning of P2 for putative cleavage sites did not reveal any sites that fit the consensus sequence established for the P1 polyprotein above. However, a related AYEE^452^/G sequence was previously identified as a putative cleavage site between the MP and CP domains (Thompson et al., 2002; Bhagwat et al., 2016). This sequence would meet the P1 consensus with the exception of the presence of a glutamate (E) rather than a glutamine (Q) at the -1 position. In addition, a DIEE^436^/G sequence was also found in the P2 polyprotein of SMoV NSPer3 and NSPer51, although this sequence is altered to DIDE^436^/G in all other isolates. We first performed a *trans*-cleavage assay using a partial P2 polyprotein precursor termed 365-735 (numbering refers to the amino acids from P2 that are included in the precursor; **Figures 4A,B**). This precursor overlapped both putative MP-CP cleavage sites (**Figure 4B**). The VPg-Pro was used as a source of active RNA1-encoded protease to be provided *in trans* and was synthesized by *in vitro* translation in the presence of unlabelled methionine. Processing of the 365-735 precursor by VPg-Pro resulted in the accumulation of two cleavage products after an overnight incubation. These products corresponded in size to the C-terminal region of the MP (14.3 kDa) and the N-terminal region of the CP (30.7 kDa) (**Figure 4B,C**, lane 2). Using mutagenesis, we investigated which of the two possible cleavage sites between the proposed MP and CP domains was recognized by VPg-Pro for processing. As above, we mutated the glutamate at the -1 position of the E/G dipeptide to an alanine. Mutation of the first putative cleavage site (A436 mutant) did not prevent the release of the cleavage products (**Figure 4C**, lane 4). In contrast, mutation of the second putative cleavage site (A452 mutant) abolished the processing (**Figure 4C**, lane 6). As a control, we also tested a derivative of the VPg-Pro that incorporated the Pro*-null* mutation. *In vitro* translation with labeled methionine confirmed that both the wild-type and mutant derivative of VPg-Pro were expressed to similar levels (**Figure 4D**). As expected, cleavage of the 365-735 precursor was not observed when incubated with the Pro-*null* derivative of VPg-Pro (compare **Figure 4E**, lane 4 to **Figure 4F**, lane 4).

Next, we tested whether VPg-Pro could cleave the P2 polyprotein at other cleavage sites. All potential Q/G, Q/S, or E/G dipeptides were considered, even if they did not entirely meet the consensus for an E or Q at the -2 position and an A at the -4 position. Dipeptides Q^314^/G, Q^949^/G, E^1412^/G, and Q^1589^/S were identified as putative cleavage sites (**Figure 4A**). In addition, since some picornavirus 3C proteases show relaxed specificity for the +1 position (Seipelt et al., 1999), we also considered dipeptides Q^875^/N and Q^1086^/T. We tested a collection of partial P2 polyprotein precursors that covered these cleavage sites (**Figure 4A**). However, we could not detect any processing events that could be attributed to the activity of a functional VPg-Pro (compare **Figures 4E, F**).

**FIGURE 5:**
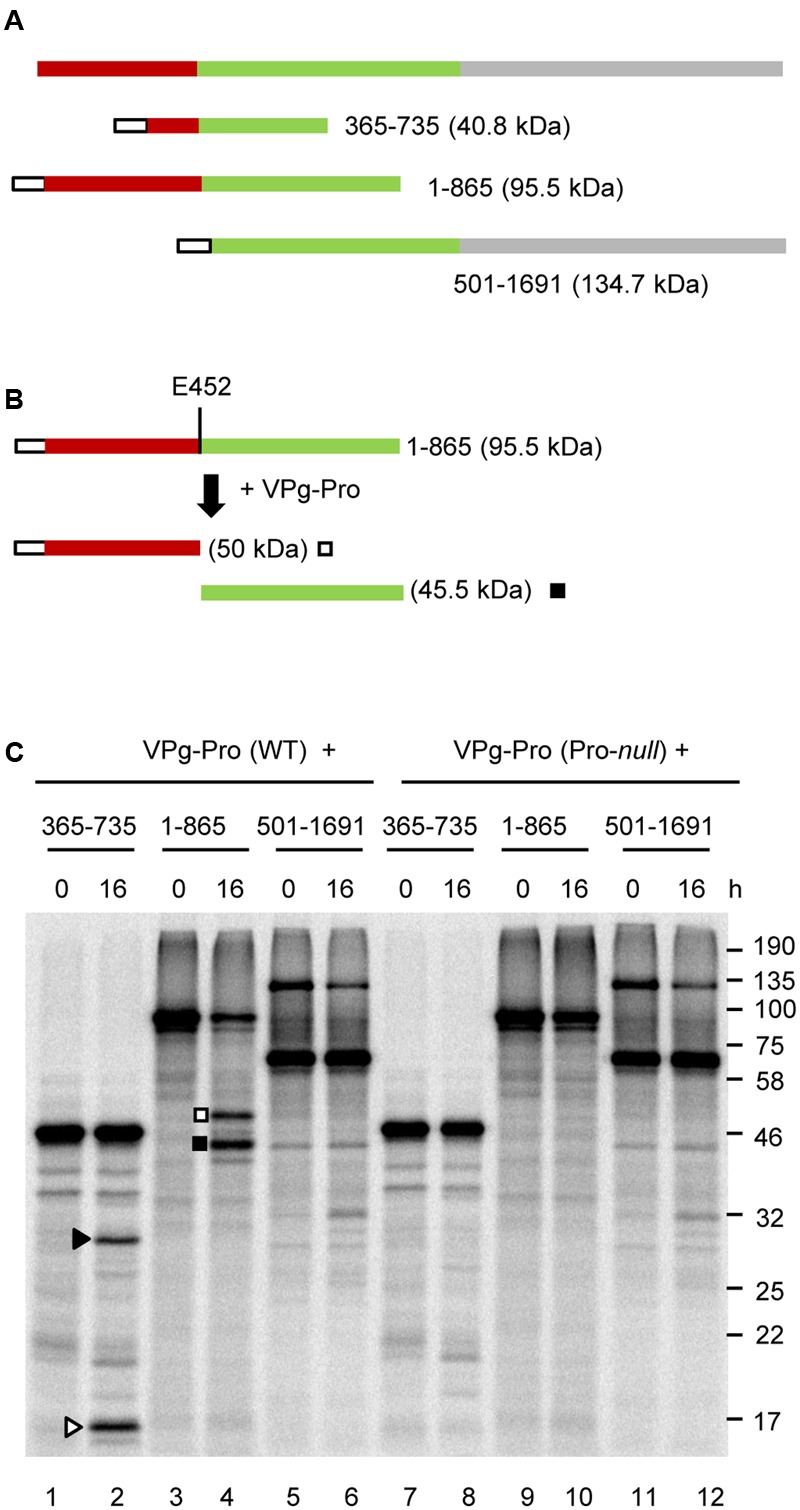
***In vitro trans*-processing assays of larger partial P2 polyprotein precursor clones confirm that the RNA1-encoded protease recognizes only the MP-CP cleavage site. (A)** A schematic representation of overlapping partial P2 precursor polyproteins is shown. **(B)** Schematic representation of the 1-865 precursor and the detected cleavage products is shown. **(C)** *In vitro* processing reactions of the P2 polyprotein precursor clones were incubated with unlabelled VPg-Pro (wild-type or Pro-*null* mutant derivative) overnight at 16°C followed by separation of the polyprotein precursor and potential cleavage products by 12% SDS-PAGE. Cleavage products are represented by symbols on the right side of each lane as shown in the schematic representations (**B** for precursor 1-865 and **Figure 4B** for precursor 365-735). The migration positions of molecular mass markers (kDa) are indicated on the right of the gel.

To rule out the possibility that the protein conformation of the partial P2 precursors may have affected the proper presentation of the cleavage sites to the VPg-Pro, we generated two larger overlapping partial P2 precursors that included either the N-terminal region of the polyprotein (1-865), or the C-terminal region starting after the predicted CP(s) domain (501-1691) (**Figures 5A,B**). Similar to the 365-735 precursor, cleavage was detected between the MP and CP domains in the larger 1-865 precursor and was dependent on the catalytic activity of the VPg-Pro supplied *in trans* (**Figure 5C**, lanes 4 and 10). However, no other cleavage events were detected in this precursor. Precursor 501-1691 was not cleaved by VPg-Pro since we did not observe a different banding pattern when the wild-type or mutated VPg-Pro were supplied *in trans* (**Figure 5C**, lanes 6 and 12). Together these results suggest that the RNA1-encoded VPg-Pro intermediate is active on a single *trans*-cleavage site over the entire RNA2 polyprotein. The results also provide an updated cleavage consensus sequence for SMoV cleavage sites of AxE (Q or E)/(G or S) (**Table 2**).

## Discussion

**FIGURE 6:**
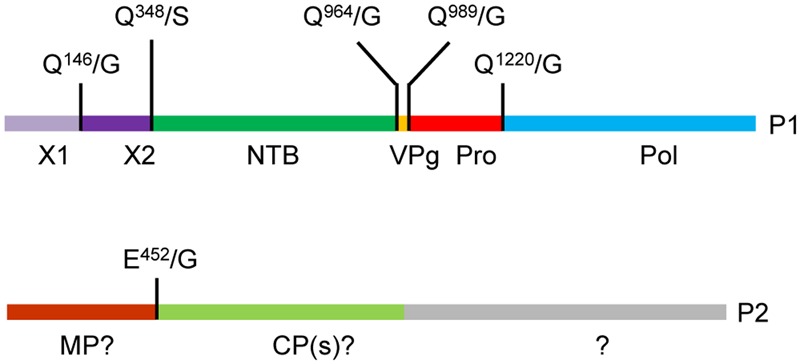
**Updated genomic organization of strawberry mottle virus isolate NsPer3.** The P1 and P2 polyproteins are depicted showing experimentally confirmed cleavage sites with vertical lines. Abbreviations and numbering are as described in **Figure 1**.

In this study, we used *in vitro* translation assays to investigate the proteolytic processing of SMoV (Canadian isolate NSPer3) P1 and P2 polyproteins. We confirmed the *cis-* and *trans-*activity of the RNA1-encoded 3C-like protease and identified five cleavage sites on the P1 polyprotein and one cleavage site on the P2 polyprotein (**Figure 6**). The results identify a consensus cleavage sequence for the SMoV 3C-like protease and help clarify the genomic organization of the SMoV RNAs.

All observed cleavage events in the SMoV P1 polyprotein occurred after a Q residue which was expected given the presence of an H residue in the substrate binding pocket of 3C and 3C-like proteases (Bazan and Fletterick, 1988; Allaire et al., 1994; Sanfacon et al., 2011). The MP-CP cleavage sequence contained an E residue at the -1 position, suggesting that it can also be accommodated in the substrate binding pocket of the SMoV 3C-like Pro, likely owing to the structural similarity shared between Q and E residues. Cleavage at E/G dipeptides has been reported for several picornavirus proteases (Seipelt et al., 1999), but has not been typically observed for the 3C-like proteases of members of the family *Secoviridae*. One exception is the protease of apple latent spherical virus (genus *Cheravirus*) which has been shown to cleave at two Q/G dipeptides and one E/G dipeptide to release the three CP domains from the P2 polyprotein (Li et al., 2000). E/G or E/S cleavage sites were also predicted for two other cheraviruses based on sequence alignments (James and Upton, 2002; Petrzik et al., 2016).

The +1 position in the SMoV cleavage sites was either a G residue (in five confirmed cleavage sites) or an S residue (one cleavage site). The stringency of requirement for a specific amino acid at the +1 position has been reported to vary with the protease due to different conformations of the substrate-binding pocket (Seipelt et al., 1999). In some cases, a G is strictly required while in other cases other small amino acids are tolerated including S, A, M, or T (Wellink et al., 1986; Carrier et al., 1999; Seipelt et al., 1999). SMoV cleavage sites identified in this study contained A and E residues at the -4 and -2 positions, respectively, with only two exceptions (**Table 2**). The X1-X2 cleavage sequence contains a C residue at the -4 position and the Pro-Pol cleavage sequence has a Q residue at the -2 position (**Table 2**). The -4 and -2 positions of the cleavage site are frequent specificity determinants for 3C or 3C-like proteases (Pallai et al., 1989; Cordingley et al., 1990; Blair and Semler, 1991; Carrier et al., 1999). For instance, the -2 position of ToRSV cleavage sites normally consists of a C or V residue and substitutions are generally not well tolerated at this position (Carrier et al., 1999). Several picornavirus 3C proteases require a small aliphatic residue at the -4 position of their cleavage sites, similar to the A residue found in most SMoV cleavage sites (Pallai et al., 1989; Cordingley et al., 1990; Blair and Semler, 1991). The -4, -2, -1, and +1 positions of the SMoV NSper3 cleavage sites (as shown in **Table 2**) were also found to be conserved in all other SMoV isolates with the exception of the NTB-VPg cleavage site of the SMoV Netherlands isolate 1134, which has a V at the -4 position (data not shown). It is interesting to note that in our study most other tested cleavage sites that did not show detectable proteolytic processing (i.e., Q^314^/G, E^436^/G, Q^875^/N, Q^949^/G, Q^1086^/T, E^1412^/G, and Q^1589^/S from P2) lacked two or more of the conserved residues at the -4, -2, or +1 positions. The possible Q^465^/G cleavage site in the P1 polyprotein was also not recognized, although the primary sequence was in agreement with the consensus sequence, with the exception of an L at the -4 position. The conformation of the polyprotein (secondary or tertiary structure) may have obstructed the presentation of the cleavage site to the protease as has been previously shown for other viruses (Ypma-Wong et al., 1988; Clark et al., 1999).

Because of the highly conserved signature motifs in the NTB, Pro, and Pol domains, the NTB-VPg, VPg-Pro, and Pro-Pol cleavage sites are confidently predicted based on amino acid sequence alignments. Deducing a consensus cleavage site sequence based on these cleavage sites (e.g., establishing preferred amino acids at the -4 and -2 positions in the case of SMoV) can assist in validating predictions for other cleavage sites. Using this principle, we examined amino acid alignments that included viruses in the family *Secoviridae* that are most related to SMoV. This included BRNV, CLVA, and DMaV (Halgren et al., 2007; Wylie et al., 2011; Bhagwat et al., 2016; Hayashi et al., 2017). These viruses also have a histidine in the substrate-binding pocket of the protease, suggesting similar requirements for a Q or an E residue at the -1 position. We did not include SDV in the analysis, as the 3C-like protease of this virus recognizes different cleavage sites due to the absence of the conserved histidine in the substrate-binding pocket (Iwanami et al., 1998; Sanfacon, 2015). Putative X1-X2, X2-NTB, NTB-VPg, VPg-Pro, and Pro-Pol cleavage sites were identified for all viruses analyzed (Supplementary Figure S2). Comparison of these predicted cleavage sites identified conserved features at the -1 position (Q or E), +1 position (G, S, or A) and -2 position (S or Q for BRNV, A or C for CLVA and L for DMaV, **Table 2**). These results suggest that the presence of six protein domains in the P1 polyprotein is likely a conserved feature amongst this group of related viruses, which is shared with nepoviruses but distinct from comoviruses and fabaviruses (Wang and Sanfacon, 2000; Wetzel et al., 2008, 2013). The X2 protein of nepoviruses shares several properties with the cowpea mosaic virus (CPMV) Co-Pro including the conserved motif F-X_28_-W-X_11_-L-X_23_-E (Zhang and Sanfacon, 2006; Sanfacon, 2013), a motif which is partially conserved in SMoV (Supplementary Figure S1). The CPMV Co-Pro regulates the activity of the CPMV protease by slowing down the processing of P1 and facilitating the processing of P2 (Peters et al., 1992). However, the nepovirus X2 protein has not been reported to influence the protease activity in a similar manner (Wang and Sanfacon, 2000; Wetzel et al., 2008). Results presented here do not support a Co-Pro role for the putative SMoV X2 protein as we did not detect significant differences in the efficiency of proteolytic processing in constructs containing or lacking the X2 domain (compare **Figure 2, 3**).

Processing of the P1 polyprotein was relatively inefficient, as evidenced by the large amounts of precursor polyprotein remaining after an overnight incubation. In contrast, proteolytic cleavage of similar nepovirus P1 polyproteins was generally more efficient using similar *in vitro* assay conditions (Wang and Sanfacon, 2000; Wetzel et al., 2008). It is possible that further optimization to enhance the activity of the SMoV protease may improve the *in vitro* assay results, however, processing was not significantly improved when different temperature or pH ranges were tested (data not shown). Alternatively, the SMoV protease may be more active *in vivo*, possibly after interaction with plant host factors. Further experiments would be required to address this question. Finally, it is also possible that the relatively low activity of the protease has a biological function, such as limiting the accumulation of mature virus proteins. Indeed, low titers of the virus have been reported in infected plants (Thompson et al., 2002). We observed that cleavage was relatively more efficient at the NTB-VPg site than at other P1 cleavage sites at least *in vitro*. Further work will be necessary to determine whether this is also the case *in vivo*.

Systematic scanning of the SMoV P2 polyprotein with overlapping constructs only allowed the detection of a single cleavage event, which was located between the predicted MP and CP domains. Based on the identified cleavage site, the predicted MP domain is approximately 50 kDa, which is similar to the MP of several other related viruses such as apple latent spherical virus (42 kDa), tomato torrado virus (50 kDa), ToRSV (48 kDa), and CPMV (48 kDa) (Wellink and Van Kammen, 1989; Wieczorek and Sanfacon, 1993; Yoshikawa et al., 2006; Verbeek et al., 2007). Cleavage sites were also predicted at corresponding positions in the P2 polyprotein of the related BRNV, CLVA, and DMaV, suggesting the presence of a similar N-terminal MP domain (**Table 2**).

We anticipated finding at least one additional cleavage site downstream of the predicted CP domain. Based on alignments with the SDV P2 polyprotein (Iwanami et al., 1999), the SMoV CP domain is predicted to be approximately 60 kDa. Yet the entire region of the P2 polyprotein downstream of the mapped MP-CP cleavage site is approximately 130 kDa. This result raises the intriguing possibility that release of the mature CP protein is regulated by a mechanism distinct from the action of the RNA1-encoded 3C-like protease. This could include processing by a second viral protease or a plant protease, or a premature translation termination event, such as the 2A-like stop-go translation reprogramming mechanisms characterized for some picornaviruses (Atkins et al., 2007; Roulston et al., 2016). We are currently investigating these possibilities.

## Author Contributions

KM, MW, and HS: Conceived and designed experiments. KM and MW: Performed the experiments. KM and HS: Wrote the manuscript. All authors read and approved the final manuscript.

## Conflict of Interest Statement

The authors declare that the research was conducted in the absence of any commercial or financial relationships that could be construed as a potential conflict of interest.
