# Corrigendum: Identification of Cleavage Sites Recognized by the 3C-Like Cysteine Protease within the Two Polyproteins of Strawberry Mottle Virus

**DOI:** 10.3389/fmicb.2017.01762

**Published:** 2017-09-12

**Authors:** Krin S. Mann, Melanie C. Walker, Hélène Sanfaçon

**Affiliations:** Agriculture and Agri-Food Canada, Summerland Research and Development Centre Summerland, BC, Canada

**Keywords:** proteolytic processing, viral proteases, plant virus, cleavage site specificity, 3C protease, picornavirales, secoviridae, *in vitro* translation

In the original article, there were mistakes in Table 2 as published. Several listed cleavage sites were derived from virus isolates that did not correspond to the accession numbers provided in the Materials and Methods Section. In addition, the sequence listed for the DMaV NTB-VPg cleavage site was incorrect and showed the sequence for the VPg-Pro cleavage site instead. Corrections to the SMoV MP-CP cleavage site, BRNV MP-CP cleavage site, and DMaV X1-X2, NTB-VPg, and Pro-Pol cleavage sites have been made. Please note that this correction does not change amino acids at conserved positions (highlighted in red or green in the Table). The corrected Table 2 appears below. The authors apologize for this error and state that this does not change the scientific conclusions of the article in any way.

**Table 2:** Alignment of potential cleavage sites for members of the family *Secoviridae*.

	**SMoV**	**BRNV**	**CLVA**	**DMaV**
**P1 SITES**				
X1-X2	AQCVEQ/GG	IGLEQE/GF	EHETCQ/GL	TEEELQ/GL
X2-NTB	CPAYEQ/SS	DVPLSE/GA	ADVAAQ/SG	EPMMLQ/AG
NTB-VPg	EVATEQ/GG	LAFTSE/GG	SSSLAQ/GT	TESELQ/GV
VPg-Pro	VRAYEQ/GA	IKPYSQ/GG	RAFSAQ/GE	RGFQLQ/GG
Pro-Pol	EVAVQQ/GM	GKFYQQ/GD	PVIVAQ/GP	PADELQ/SE
**P2 SITES**				
MP-CP	TRAYEE/GXF	DDFVEE/GG	GDAAAQ/GD	LNDSLE/GD

*Amino acids highlighted in red represents highly conserved positions. Amino acids highlighted in green represent alternative amino acids found at the −4, −2, −1, or +1 positions. SMoV, Strawberry mottle virus; BRNV, black raspberry necrosis virus; CLVA, chocolate lily virus A; DMaV, dioscorea mosaic-associated virus; NTB, nucleoside triphosphate binding protein; VPg, viral genome-linked protein; Pro, protease; Pol, polymerase; X1 and X2, unknown; MP, movement protein; CP, capsid protein*.

## Conflict of interest statement

The authors declare that the research was conducted in the absence of any commercial or financial relationships that could be construed as a potential conflict of interest.

