# Supplementary material for: Identification of Cleavage Sites Recognized by the 3C-Like Cysteine Protease within the Two Polyproteins of Strawberry Mottle Virus
Source: Front Microbiol. 2017 Apr 27;8:745. doi: 10.3389/fmicb.2017.00745 (PMC5407059; doi:10.3389/fmicb.2017.00745)
Supplement: Supplementary file 1 [file Image_1.PDF]

Supplementary Figure S1: Multiple sequence alignment of the P1 amino acid sequences from strawberry mottle virus (SMoV), tomato ringspot virus (ToRSV), and arabis mosaic virus (ArMV). Alignment was generated using Clustal Omega (version 1.2.4). Cleavage sites confirmed to be cleaved by *in vitro* translation assays are highlighted in red, while a previously predicted cleavage site is highlighted in blue. Highly conserved motifs in the X2, NTB, Pro and Pol domains are highlighted in green.

```
SMoV_NSPer3         ------------------------------------------------------------      0
ToRSV_Rasp2         ------MSSICFAGGNHARLPSKAAYYRAISDRELDREGRFPCGCLAQYTVQAP------      48
ArMV_NW             MWQISEGSQCCCTGKTWS--------------NAEAKEARYVCNCILSCRLVKVEVVPQL      46

SMoV_NSPer3         ------------------------------------------------------------      0
ToRSV_Rasp2         -----PPAKTQEKAV--------GRS-----ADLQKGNVAPLKKQRCDVVVAVSGPPPLE      90
ArMV_NW             PKSRIAPAQDKAERITPLCNSNGGAAPTIPKSKRAFEPRTPLIKQRCDVVVRVGPPADLD      106

SMoV_NSPer3         ------------------------------------------------------------      0
ToRSV_Rasp2         LVYPARVGQHRLDQPSKGPLAVPSAKQTSTAMEVVLSVGEAALTAPWLLCSYKSGVSSPP      150
ArMV_NW             LVYPALVQEEVAIPPTEKVL-QPTLKA--------------EV-RVPIFCAPKRMVAFPK      150

SMoV_NSPer3         ------------------------------------------------------------      0
ToRSV_Rasp2         PPMTQRQQFAAI-----------------KR-RLVQKGQQIIRELIRARKAAKYAAFAAR      192
ArMV_NW             PPTKIASKRDALQFPAGAVAFNGINFIDAKGKVVLSEGAKRILKGIRVAKQQR--QRTAR      208

SMoV_NSPer3         ------------------------------------------------------------      0
ToRSV_Rasp2         KKAAAVAAQKARAEAPRLAAQKAAIAKILRDRQLVSLPPPPPPSAARLAAE----AELAS      248
ArMV_NW             RSAACKKVRKAR--------DLALFKRLSEECTFQDLPGG---FAGEIPAGHACYRKVAA      257

SMoV_NSPer3         ---------------------------MSYLCDDVAMWIDTFTQLG-----MPLDMAMDV      28
ToRSV_Rasp2         KSASLQRLKAFHRANRVRPVLNNSFPSPPLACKPDPALLERLRLATPSRCTVATKRQRDF      308
ArMV_NW             PTTSFKK--EVSKGKKAKKPSTPVLPAQDFSCVDSFDWGEK------S---SPVEIEDDW      306
                                                   *       :              .   *

SMoV_NSPer3         YMRTKINDLRK-------------NPVALSACYTYKEWVDEHFVTQAPI-DD---DYVP-      70
ToRSV_Rasp2         VVAPLATQIRVAKCASHQ------EAYDSCRSILIEEWPESRYLFGPLSFVGDWEHV-PG      361
ArMV_NW             VLIEKPVLQRQAAHSAQGRATEALTRFAASGGFTVKAHQK----VEELASSGEAGHLIAG      362
                     :       *                   .     :   .           .   .

SMoV_NSPer3         -----------TEVFPDEPPVSEVLPEYPKAGADIALVDIVTDQLYSSPIFPLTEVKCRV      119
ToRSV_Rasp2         MLMQYRLCVLFSMVRDVMPALSLVA-----------------DTLH--------ALRSGT      396
ArMV_NW             EFA--ELCL-RSLVYNDAPVLSASI-----------------EELI--------------      388
                               : *    * :*                    : *

                                         X1-X2 cleavage site
SMoV_NSPer3         LASRDINQLSRLADAVTAVTVAQCVEQGGISVGGFQEAISSRIR---RSLGWVGKVVESV      176
ToRSV_Rasp2         A----PNIVFKNAMSTANQ-ILECSH-SSHAAQGFGNFLSRGKSAAINLASGLSSFVGEK      450
ArMV_NW             -----TEQDFKDAIELFNIELAELPT-DSTTCGQFNDWAS----AAKKMAKGVGSIVGDF      438
                          :   : *       : :    .. :   * :  *       .    :...* .

                                             “Co-Pro” motifs
SMoV_NSPer3         NNMASATALLNKIFERILSSFD--FIAQTVSLFSDFLKGMKEKL---MEMCLKTWEKLAE      231
ToRSV_Rasp2         VVS-GANHVVNKASEVIVDKLFVPFVKLLREHFDDTIGKWIPKLLGATQKIEELWRWSLE      509
ArMV_NW             ARMSGAGVLIT-F-DRCIEYLQKKALTFCQKVFNATMAPYLSHLAEASNIISKIWKKLAE      496
                        .*  ::.   :  :. :    :    . *.  :     :*    :   : *.   *

SMoV_NSPer3         WGEHFYYVIPMFCSIF----L-------IATTCFLINKFLSV--VAPRYCFSSSSIIQLI      278
ToRSV_Rasp2         WAQNMSKKLDVSLRVLRGSALVGVGLLLVSGILYFAEQLLRSFGLLI----VAGSFISMF      565
ArMV_NW             WMESLKGKAGLALEVLAQHAIFALGAIVVGGVVVLVEKVLVACKVIPNCGIVLGAFLTLF      556
                    * : :     :   ::    :       :.    : ::.*    :        .::: ::
```

```
SMoV_NSPer3      VVGCAIVGCKEMGAALLA--LSSAGKKSFLDLIYETFGVDTSEFSDTLPKDENAVPSPAD       336
ToRSV_Rasp2      VGGCLLAYAGSMAGIFDEQMMRVRGILCEIPMLLYLKAQPDPFF----PKKSG-------       614
ArMV_NW          FASLGLTALE----CTAEEIFRMHQ-CCK-GAIYSMYSVKEPM------NEAE-------       597
                 . .  :.              :      .    :    .           :.

                      X2-NTB cleavage site
SMoV_NSPer3      WSQFENCPAYEQSSTSMTGFFGILG----LLTFFAPRGMK---CDLYEMTKWAHGLKGLA       389
ToRSV_Rasp2      ------------GRAPTQGLTDVFGVPLSIMNAIGDGLVHHSLDTLTLMGKFGAAMDNVR       662
ArMV_NW          ------------GSSVTMGVLQGLDNAISALTRVGQSMISFKLGSFSYYAKIAQGFDQLA       645
                             . :   *.   :.     :. ..   :      :    * . .:. :

SMoV_NSPer3      DGYEKFKSITEKLAFWVYERIGLDTTWD----APAIQSMILVTGIRFQDWCSEVEKLSID       445
ToRSV_Rasp2      KGITCMR----SFVSWLMEHLALALDKITGKRTSFFRELATLINFDVEKWVRDSQQYLLA       718
ArMV_NW          RGKKAIG----ELTGWLIDLVGGVYSKVSGQESTFFDELSTIVCLDVRSWLLKSKRVRLQ       701
                  *   :     .:. *: : :.          :  : .:  :  : ...*  . ::  :

                             Previously predicted
                                cleavage site
SMoV_NSPer3      MLNYTNL-QEDLTRARKLKEQGDKIQTHMMYS---TESISFMMREKLRASLTTIASVIAK       501
ToRSV_Rasp2      AEIYVDGDTVVMDTCRHLLDKGLKLQRMMVS---AKSGCSFNYGRLVGDLVKRLSDLHKR       775
ArMV_NW          VETMAIGDRITLDTISKPT--GMQGHKILITAAGVPRKTSADFTMCIKEEVSKLEEVHQR       759
                     .      :    :    * : :  ::         *      :   :. : .:  :

                                  Helicase (or NTB) motifs
SMoV_NSPer3      FEKAVDISGTRMCPFTVLFHGPAGSGKSNSMRGFMHDVMNEMGEPSVGRIYPRNSGDKHW       561
ToRSV_Rasp2      YCASGRRVHYRLAPFWVYLYGGPRCGKSLFAQSFMN-AAVDFMGTTVDNCYFKNARDDFW       834
ArMV_NW          TACAGINEGMRQFPFPWVYIFGASQSGKTTIANSVIIPSLLEEMNLPKTSVYSRPKTGGFW       819
                    :      *  ** * :.*   .**:   ...:     :         * :   . .*

SMoV_NSPer3      SGYLRQTALYYDEFAQKKPTNGESDELELIPLVSCSHFPLFGAAIEDKGLSFNSKYVFMC       621
ToRSV_Rasp2      SGYRQEAICCVDDLSSCETQ--PSIESEFIQLITTMRYGLNMAGVEEKGASFDSKMVITT       892
ArMV_NW          SGYARQACVKVDDFYAIEQT--PSLASSMIDVVNSEPYPLDMAYLHEKGMSMDSPLVVTT       877
                 *** :::    *::   :     *   .:* ::.   : *  * :.:** *::*  *.

SMoV_NSPer3      SNRADVSPNAGLADNDAFRRRRHLCVEVTRDA-REFDPSNPTYNQTFQLKN---PLKPTE       677
ToRSV_Rasp2      SNFFTAPTTAKIASKAAYNDRRHACILVQRKEGVAYNPSDPAAAAEAMFVDSTT------       946
ArMV_NW          ANTVKPPTNAGITDEASFFNRRAAVIEVRRKDNTHFTPRAYDNCIEVRFLHNKCAYVDSE       937
                 :*      .* ::.: ::  **   : * *.    : *          : .

SMoV_NSPer3      NLKFSRDGGAPEEVGPMSYNELVIYAVNRAQEHFDRETKAMKYAVSRATNTGRAHEQAIY       737
ToRSV_Rasp2      --------QHP-LSEWMSMQELSAELLLRYQQHREAQHAEYSYWKSTSRTSHDVFDILQK       997
ArMV_NW          GIPQGPAVNTPMEEGWISPSEAVATLKNLLGEHVLAEEEKLLDYRERIGNDHPIYNAAQE       997
                           *     :* .*          :*   :        .   .    .:

SMoV_NSPer3      YCP--RFTCERMKLDTHACPHLANEHFQGHEVYGEFRGDYFCCDKNGTA-------CDCP       788
ToRSV_Rasp2      CV---NGDTQWLSLPVDVIPPSIRQKHKGNRVFA-IDGRIFMFDYMTLEYDEIKEKENLD       1053
ArMV_NW          FIGNMHYPGQWLTTEQK---NTYGINEEGFSFLA-VDGKMYKYNVLGKL-------NPCE       1046
                      .   : :.   .        : :*  . . . *  :  :

SMoV_NSPer3      LTNWEKSIMHDMSANATDDEIALALALFSQ-------EENRLMSDYAGFFELIDDMDNW-       840
ToRSV_Rasp2      ARHLEARILEKYG------DTRLLLEKWGA---------NGVVAQFIE--QLLEGPSNVA       1096
ArMV_NW          TVPPHPNVIPWLE------EKTLSIVHWDAHKHIATGPRNALVSCFLQ--GLVQDQSRVQ       1098
                     .  ::          :  * :  :.          * ::: :     *::. ..

SMoV_NSPer3      RLDTPPRAKKADLQAYVNRTWADYNDRLRFLMCQHF-DRTKSAR-----NSYFQRLKTLK       894
ToRSV_Rasp2      SLEVLSKDSLESHKEFFSTL--GLIERATLRAVQKKIDAAREDLMHLSGLKPGRSLTELF       1154
ArMV_NW          SVDLMGKDSSPEQQAFFKRL--TLSERIYLRLCQIRIDAVKKEQLSSVSRGALDVL----       1152
                  ::   : .  . : :..       :*  :   *   * .:.             *

SMoV_NSPer3      DDIRSWSVVGAWNTLPM--GAKWLVGIIALFSFGASLIWLLSKVMAMHTWNPMEMLGVFL       952
ToRSV_Rasp2      VEAYDWVYANGGK------LLLVLAAVILILFFGSACI-KLMQAIFC-GAAG-GTVSMAA       1205
ArMV_NW          ---RDCMYKSKAKLVENYSLLLTLVAILVLIATAYSLISTLIGLAGCSSFAG-GMVALNH       1208
                     .    .  :          *..:: ::  . : *  *              :.:

                      NTB-VPg                      VPg-Pro
                    cleavage site               cleavage site
SMoV_NSPer3      GSRSFIEVATEQGGYAESGSN-TQAPIYRHKRVRAYEQGASDSQVMDLNDSEKIEAIKKA       1011
ToRSV_Rasp2      VGKMTVQSTIPSGSYADVYNARNMTRVFRPQSVQGSS--LAEAQ---FNESHAVNM----       1256
ArMV_NW          VSNASIPCSEPR--LEEGYIPRNKF-VSRISRTRGDG--PAQGQ---GDHEELVTE----       1256
                  ..  :  :       :     .   : * . .:.     ::.*    :... :
```

```
                                  His of protease catalytic triad
SMoV_NPer3       QGILVFSKNDGKSAAAAVTIFKDHQFLITTHELALLNFSKGCI-----------------       1054
ToRSV_Rasp2      ----LVRIDLPDGNIISACRFRGKSLALTKHQALTIPPGAKIHIVYTDNNGNTKAPLTHF       1312
ArMV_NW          ----LYYYFDGVKRLISCCWFKGRSLLLTRHQAMAIPIGNEIQVIYADGTERKLVWP---       1309
                     :           :   *:.:.: :* *:   :  .

SMoV_NPer3       --LTMRSGASYAIYIDAGNVVVGSKGGIK-DPISVVKVSTYFG----------M-AKACT       1100
ToRSV_Rasp2      FQPTGPNGEHFLRFFNGTEVCIYSHPQLSALPG---APQNYFLKDVEK-ISGDIAIKGCG       1368
ArMV_NW          GRQEDRSCKGYI-EFPDNELVVFEHARLLTMPI---KYEKFFVDDPDHQISPNVAVKCCV       1365
                       .   :   :   :: : .:  :   *      ..:*           :  * *

SMoV_NPer3       GSIMFDFGTYCEGHHNGIVIPNAHKSLDQSVLKSTFSRRHEVIDIHNDKGRVVWQANNLM       1160
ToRSV_Rasp2      IKLGRTSVGECVGVKDNEPVLNH--------WRAVAKVRTTKITIDNYSEGGDYSN-DLP       1419
ArMV_NW          A-----------RLEDGIPQFHF--------WNKYASARSDVHTIKDEGGSAVYQN-KIR       1405
                               .:.    :           .   . *     *.:      :.  .:

                       Cys of protease       His or Leu of protease
                       catalytic triad       substrate-binding pocket
SMoV_NPer3       TVPIY---HQVGHCGRLLLARDEAKCLKIVGVHVAGIVIQE--------KYISLFSEINA       1209
ToRSV_Rasp2      TSIISEYVNSPEDCGALLVAHLE-GGYKIIGMHVAGSSYPVEVDGVQMPRYISHASFFPD       1478
ArMV_NW          RYIIYAHEAKRNDCGAIAVAEIQ-RTPKVLAMLVSGIGNVT------------YSSVIPS       1452
                    *     .  .** : :*. :    *::.: *:*                  * :

                    Pro-Pol cleavage site
SMoV_NPer3       IHKSAEVAVQQGMDIDILELVEPETKTEMVVKIGQVAHGQQF-RPASRTAIIKSQ-IHDT       1267
ToRSV_Rasp2      YSSFAPC--QSS--VIKSLIQEAGVEERGVSKVGHIKDPAETPHVGGKTKLELVDEAFLV       1534
ArMV_NW          YSSSF-----VRG--DVPYVPEDGIKTNGYRKVGYLMA-KDAPHVPSKTAFMKVPDEICF       1504
                   .               : *   : .   *:* :    :  :   .:* :

SMoV_NPer3       LWRAPETEPTVISP-TDPRVPYQ---FDPYTAGIMKFEKEVGPLDFTDPDSHESTVVQDI       1323
ToRSV_Rasp2      PSPVEVKIPSILSK-DDPRIPEAYKGYDPLGDAMEKFYEPMLDLD---EDVLES-VMADM       1589
ArMV_NW          PYPNP-KQPAILSAEDERLIGTVHEGYTPIREGMKKFAEPMHLLD---AQLLDE-VAGDM       1559
                       . *:::*   :  :       : *   .: ** : :  **    :  :. *  *:

SMoV_NPer3       TEELAREKKAIGGFALDTVCSDDVAINGVEGVPYAERLVMSTSEGYPFVLSRKAQDTGKF       1383
ToRSV_Rasp2      YDEFYDC------QTTLRIMSDDEVINGSDFGFNIEAVVKGTSEGYPFVLSRRPGEKGKA       1643
ArMV_NW          VHTWFDA------GEILEDVPLSIAINGDVEEEYFDPIAMDTSEGYPEVLQRKNGEKGKA       1613
                  .                    . .***       : :. .****** **.*:  :.**

                                                       RNA-dependent RNA polymerase
                                                                motifs
SMoV_NPer3       RFFDKDGD---------KWIAKDEVLDDLHELEESIKSEDFSGGIITIACAKDEKTKIKK       1434
ToRSV_Rasp2      RFLEELEPQPGDTKPKYKLVVGTEVHSAMVAMEQQART--EVPLLIGMDVPKDERLKPSK       1701
ArMV_NW          RFFVGE---PG----AREFVPGCGPERAYLSLEEECKT--RIPSLVSIETPKDERLKRSK       1664
                 **:              : :             :*:. ::      :: :   ***: * .*

SMoV_NPer3       VREVPKTRIFEILPFHYNILVRKYYLFFMQFIMSLHDFLPCKVGLNVYSKSWDTMHA-EH       1493
ToRSV_Rasp2      VLEKPKTRTFVVLPMHYNLLLRKYVGILCSSMQVNRHRLACAVGTNPYSRDWTDIYQRLA       1761
ArMV_NW          -IETPGTRLFSVLPLAYNLLLRVKFLSFSRLLMKKRSHLPCQVGINPYSREWTDLYHRLA       1723
                   * * ** * :**: **:*:*     :   :   :  * * ** * **:.*  ::

SMoV_NPer3       NRFAYHFNGDYTGFDTATPRVLMMRIADMVSELACDGRENAIVRRNLMRMAVERRILVLR       1553
ToRSV_Rasp2      EKNSVALNCDYSRFDGLLNYQAYVHIVNFINKLYND--EHSIVRGNLLMAMYGRWSVCGQ       1819
ArMV_NW          EKSDVGYNCDYKGFDGLITEQILAVVATMINAGFRN-PVSNQQRSNLLMAISGRLSICGS       1782
                 ::      * **. **           :. ::.    :       * **:     *  :

SMoV_NPer3       DLYQVKGGTPSGFALTVIINSVVNQFYLMWAWRKIMSRIDPGLVPYRVMRSHCTFSVYGD       1613
ToRSV_Rasp2      RVFEVRAGMPSGCALTVIINSLFNEMLIRYVYRITVPRPLVN-----NFKQEVCLIVYGD       1874
ArMV_NW          QVYETEAGIPSGCALTVVINSIFNELLMRYCYKKIVPPIYRE-----CFDRCVVLITYGD       1837
                  :::...* *** ****:***:.*:: : : :: :   :             :     :  .***

SMoV_NPer3       DNVVSFSLQVKDMYNLVTIASELKVIGVNLSDGKKTGN-LVKWMDFSDLDFLKRRWVLSS       1672
ToRSV_Rasp2      DNLISIKPDTMKYFNGEQIKTILAKYKVTITDGSDKNSPVLRAKPLKQLDFLKRGFRVES       1934
ArMV_NW          DNVFTVSQSIMTSFTGDALKAEMANLGVTITDGKDKSLATIPARPLLELEFLKRGFKKGN       1897
                 **:.:.. .    :.    : : :    *.::**....   :     : :*:**** :   .

SMoV_NPer3       GQGFLCPLDKSAIEERLFWVRTSED-SVETLDDNCYSALMEAFHHG-REYFQFLRTKIQD       1730
ToRSV_Rasp2      DGRVLAPLDLQAIYSSLYYINPQGN-ILKSLFLNAQVALRELYLHGDVEQFTAVRNFYVN       1993
ArMV_NW          GGLIYAPLEKLSIMSSLVYIRSDGSDMLQKLVDNVNTALVELYLHQDREYSESVRDFYLE       1957
                 .  . .**:  :* . * ::. . .  ::.*  *   ** * : *  *   :*    :
```

```
SMoV_NSPer3      AYDKAGLFQP-----HLLHFNEAQ----AIWLEQHSVEP-----INDYLDGIKKEVLPLT      1776
ToRSV_Rasp2      QI-G-GNFLSLPQWRHCASFHDEQYSQWKPWSPVKFLEVDVPD--AKFLQH--------K      2041
ArMV_NW          KLPP-GSYKELTTWYEAQIFHECQLSGESGWKPQGLIEVSHGASFASFVQQ--------N      2008
                      * :       .   *:: *      *     :*        .:::         .

SMoV_NSPer3      AGRDVLRVITPTIDAVSVKGYNRVHRELAYRRTKVFLDPKAKEIMWLRSD---QAAHLNI      1833
ToRSV_Rasp2      APATALSIVADRL-AVAGPGWRNKDPDRYLLVSLTSL-KANEGGLYFPVDYGEGTGQQAT      2099
ArMV_NW          GTELERHDICPGL-AISGSKYIAREEE--ILMSLSSLLPGDINAVKLTLKCGDGIGRLPS      2065
                 .       :   : *::   :   . :     :   *       : :  .     .:


SMoV_NSPer3      PAS-LNKRNFEGIARD-VAKLLRGEKCCI-VEGTCGINSFALAL----AIGFLRKELTSV      1886
ToRSV_Rasp2      EASIRAYRRLKDHRVRHMRDSWNEGKTIVFRCEGPFVSGWAAAISFGTSVGMNAQ-----      2154
ArMV_NW          KASVLSQRKP-GIVMQLCARAIKEKKTLVIRDERPYIGGWAMACICGESFGFSIK-----      2119
                  **    *.  .          .  *  :       :..:* *     :.*:  :

SMoV_NSPer3      GCVNLLASYSSS--------DASYYTGLG---------------------------MLTA      1911
ToRSV_Rasp2      ---DLLINYGIQGG-AHKEYLGRYFVGARFKELERYDRPFQSRIIAS-------------      2197
ARMV_NW          ---DTLALYANLMGPNRKNGLATYFTD--------FDSPVHVKKIHAITNGEEGVAMLKD      2168
                    : *  *.           . *:..

SMoV_NSPer3      VLG---------------------------------------------------------      1914
ToRSV_Rasp2      ------------------------------------------------------------      2197
ArMV_NW          SFAFCEPTTIAATSCDTRKEMVSHLPTSFPNIVLIGGISYPKEGGEPGALYSPTDVVMSK      2228

SMoV_NSPer3      --------------------------------------------------------  1914
ToRSV_Rasp2      --------------------------------------------------------  2197
ArMV_NW          KLQGVYVSEAVLKCCLRCPGAAVKTVLQTSSPGSSLSQAHFRSLRRVQSHRCMRKS  2284
```
