# Supplementary material for: Identification of Cleavage Sites Recognized by the 3C-Like Cysteine Protease within the Two Polyproteins of Strawberry Mottle Virus
Source: Front Microbiol. 2017 Apr 27;8:745. doi: 10.3389/fmicb.2017.00745 (PMC5407059; doi:10.3389/fmicb.2017.00745)
Supplement: Supplementary file 2 [file Image_2.PDF]

Supplementary Figure S2: Multiple sequence alignment of the P1 amino acid sequences from strawberry mottle virus (SMoV), black raspberry necrosis virus (BRNV), chocolate lily virus A (CLVA) and dioscorea mosaic-associated (DMaV). Alignment was generated using Clustal Omega (version 1.2.4). Cleavage sites confirmed to be cleaved by *in vitro* translation assays are highlighted in red. Highly conserved motifs in the NTB, Pro and Pol domains are highlighted in green.

```
SMoV_NSPer3      MSYLCDDVAMWIDTFTQLGMPLDMAMDVYMRTKINDL----RKNPVALSA-CYTYKEWVD       55
BRNV_Alyth       MSYLTKDLAAFIESAIATGYSFDNACELYQRFKIVEL----NANPVSRAF-CRTYKEWVD       55
CLVA             -----------------------MSL--MEKLNFANLKQHLGENPSEAEILQYQYD-C--       32
DMaV             -----------------------MAC--VQPPIFNNI-----------------------       12
                                         :        : ::

SMoV_NSPer3      EHFVTQAPIDDDYVPTEVFPD----EP------------------------PV-------       80
BRNV_Alyth       EQFTIPTPEDIDILTVDTVDPLTAAEPNHSEAEDVDTDSCDSCDSADGPSSPISVLRSPL       115
CLVA             ---------IDSIQTLPGFPII-----------------------------KEAILRNRD       54
DMaV             ---------NDHIRYIGNFHLV-----------------------------KKSLLREPD       34
                                   .

SMoV_NSPer3      -------------------------SEVLPEYPKAGADIALVDIVTDQLYSSPIFPLTEV       115
BRNV_Alyth       EGFD--------VDIGEANKSLGIDVSAYPCLPKGKENCDLKNIMAGHLFSSPIFPISAI       167
CLVA             LITE-------DSDPQDLDE-----AMLYLAVTHRSIMPALHDADATRRIMSGYFVTGMF       102
DMaV             EAKPCELILQSVKDSGNLNN-----V---LELPTESVVTILDDKKWFEKSAEQEKVAEVI       86
                                                         * :    .   .       .

                                        X1-X2 cleavage site
SMoV_NSPer3      KCRVLASRDINQLSRLADAVTAVTVAQCVEQGGISVGGFQEAISSRIRRSLGWV-GKV--       172
BRNV_Alyth       KHEILAEKDLNRLASLFDVVNTVTHSIGLEQEGFSLSSLTGAIKSRISGATGLA-ASM--       224
CLVA             DDD-IAV-EHE------------------TCQGL----MDGACFELGRNLAGKLIAKFCA       138
DMaV             HPDVVKVKTEE------------------ELQGLFSNVATGFLISAGQRTFGYV-ADK--       125
                 .   :     :                                 .      *   ..

SMoV_NSPer3      -VESVNNMASATALLNKIFERILSSFDFIAQTVSLFSDFLKGMKEKLMEMCLKTWEKLAE       231
BRNV_Alyth       -VDIVANLSSASSLLEKIFQRIITAFDYIASSIGTFGTFLTGIKEKIIEKCTALWEKLAE       283
CLVA             AAPSYSSLGSIFSKFDQVLDRILSAFDWIAGVVENSCAVLSAIRAKVTAFFKNAFNKLGD       198
DMaV             CLSRYSSVAGLLEKLNKVVDKILDGFDWLSGLIDNVSSIFSAIKNRVMEFISKALEKIQN       185
                       .:..    ::::.::*: .**:::  :     .:..:: ::        :*: :

SMoV_NSPer3      WGEHFYYVIPMFCSIFLIATTCFLINKFLSVVAPRYCFSSSSIIQLIVVGCAIVGCKEMG       291
BRNV_Alyth       WGDHFYYIIPTFCAVFLVSITCFLINKFLAAVAPTYCFSMSVIVHLIVVCCALVGCKELG       343
CLVA             ILQHFSYVMPLIFGSLLTACVFYLVNRIIGCFSPEHQMSSARLIEIIAGVCCIIGVKEFG       258
DMaV             LLNHFSYLVPLVCGILIASNVFFLVNKVLQIFAPAAALNSFRVTELLAVVGAVIGVKELT       245
                   :** *::* . . :: : . :*:*:.:  .:*   :.   : .::.   .::* **:

SMoV_NSPer3      AALLALSSAGKKSFLDLIYETFGVDTSEFSDTLPKDENAVPSPAD------------WSQ       339
BRNV_Alyth       AALLSLTKAGKLNFLNMIYSTFGAEAGEANITVDDKQPST-FHGV------------DGV       390
CLVA             CALLMMTKRDKTDFLNLIKRFFGCDDESADFSGDP-A-------------------NLDE       298
DMaV             SYLLSLTKNDKRYFVNTIRNFLGVDAEVAGLVAAE-TSALPMTGAQARNEREQDLISFGS       304
                 . ** ::. .*  *:: *   :* :    .                            .

                  X2-NTB cleavage site
SMoV_NSPer3      FENCPAYEQSSTSMTGFFG-ILGLLTFFAPRGMKCDLYEMTKWAHGLKGLADGYEKFKSI       398
BRNV_Alyth       FEDVP-LSEGASSMTGFFG-VLGLLTFFTPKELKFDLYEMTKWAHGLKGLADGYEKFKVV       448
CLVA             AA--DVAAQSGVADLGFFAATVGLISFFMDGQSKNAFLRVAYASSFIKTGVDSYEKCSKM       356
DMaV             FE--PMMLQAGIFDVGIFGAVVSMISLFMDAENKSWLQKLSYSSSVVKNVSDGYDKCSKI       362
                         :..    *:*.  :.::::*     *  : .::  :  :*   *.*:* . :

SMoV_NSPer3      TEKLAFWVYERIGLDTTWDAPAIQSMILVTGIRFQDWCSEVEKLSIDML-NYTNLQEDLT       457
BRNV_Alyth       AEKLAFWAYEKVGLAATWDAPAIQSMILVTGIRFQDWCKEVDLLAVEMQ-GSASLQEDLS       507
CLVA             MSSLSTWLFSYLGRIDAQYSGAAQALLIHTGIGVTEWIDNCEKLLVEGNSTTRHMQDIMS       416
DMaV             VAQVSDWLYTKLGASSTHYAGAAQALLIHSGVGIHDWLEECEQLLTVGNTSLLTIEEMLT       422
                   .:: * :  :*   :  : * *:::: :*: . :* .: : *          ::: ::

SMoV_NSPer3      RARKLKEQGDKIQTHMMYSTESISFMMREKLRASLTTIASVIAKFEKAVDISGTRMCPFT       517
```

```
BRNV_Alyth       RARKLKEQGLKIQDYMVRGDSSISFMMRERLKATMGVINDIVSKFEKAIDIGGTRMCPFT       567
CLVA             EARTLVDQGKTISQYLMQNDSGANYIFRQKFVAVEKQLNDFYTSLKKSNLCNKYRATPFV       476
DMaV             RCRKLIDQSTKITQFLMRSEEGSTFILRHKFLAVDKQLREFYNKIVQSNMTNIFRETPFV       482
                 ..*.* :*. .*  .:: . .. .:::*.:: *    :  ..   .: ::   .   *  **.

                                Helicase (or NTB) motifs
SMoV_NSPer3      VLFHGPAGSGKSNSMRGFMHDVMNEMGEPSVGRIYPRNSGDKHWSGYLRQTALYYDEFAQ       577
BRNV_Alyth       VLFFGAPGVGKSNTMGPFMHEIMDRNGESKIGRVYPRNSGDEHWSHYMRQTALVYDEFAQ       627
CLVA             ITFMGGPGTGKSTMARPFAEAFLDEMGESKVDRIYSRNGGDAYWSNYTRQPMVVFDDFGQ       536
DMaV             IVLHGQPGVGKSTLMRVLGNDLLDQLDEPQKDRFYSRNSGDAYWSGYIRQPMVLFDDFAQ       542
                 : :  *   *  ***.     :  . .::. .*  .  .*.*  **.**  :**  *  **   :  :*:*.*

SMoV_NSPer3      KKPTNGESDELELIPLVSCSHFPLFGAAIEDKGLSFNSKYVFMCSNRADVSPNAGLADND       637
BRNV_Alyth       RTPTPGRSDELELIPLVSCNHYPLVGAAIADKGLSFNSKYIFMCSNRADVSAGAGLADPD       687
CLVA             TKQENGRFDEENLIQLVSCNPYMLPMAAVEDKGRPFDSKYMILCTNRTYANPGADLASED       596
DMaV             IQQTNGMFDEASLIPLVSCNPYLLPMAALEEKGRPFDSKYMILCTNRKKVDERCELADRE       602
                      *   **  .**  ****.  :  *    **:  :**    *:***:::*:**    ..   .  **.  :

SMoV_NSPer3      AFRRRRHLCVEVTR-DAREFDPSNPTYNQTFQLKNPLKPTENLKFSRDGGAPEEVGPMSY       696
BRNV_Alyth       AFRRRRHVCVEVFKNDNLPFRPDEPYYNQMFQLRNPLRPKDPLTYIDEAGHQQPYPRMTY       747
CLVA             AFLRRRHLVFEVHQNPDVPYNPAACYDNLLFTVKDSLRPMMEK--------SPDTTGMNF       648
DMaV             AFYRRRHVFWDVTRNADIPYDPICCHANLRFTLRNSLDADLAV--------DDTAINLGY       654
                 ** ****:   :* :      :  *      *   *  :::  *                  :  :

SMoV_NSPer3      NELVIYAVNRAQEHFDRETKAMKYAVSRATNTGRAH--------------EQAIYYCP--       740
BRNV_Alyth       GELCLYVAEKSAEHFDKEAQSLKFMRKVQGLQEQ----------------EEFVQEGPLT       791
CLVA             FEAIAFAVNTATAFREKESEALRNIGR--NFSHEFVRVDGQLRTRVPRARIEMLAEEPIA       706
DMaV             YEMLAYTANKAEEHTNRKKAALRSLAATRNSHSRVVRQDGVFRVI----------DELQS       704
                  *    :..:  :    .  :::   :::            .

SMoV_NSPer3      ----------RFTCERMKLDTHACPHLAN---EHFQGHEV-----YGEFRGDYFCCDKNG       782
BRNV_Alyth       DF------HGSLIISPKAISEISMPHFHN---T-DDNHQI-----HGCTNHKVFCCSLFG       836
CLVA             AQAGVGEASYLFSVERIVSQGGHFPNFAELVETTRRNNELGLVDTGYEFYSTDFVCDQDG       766
DMaV             DNFMLAAATSIFSLDKFLSQGGDLPNFRRMVNPADGQLD--FFWLGFKA----FFDMTGN       758
                            :   .    .    *:: .         :              *      .

SMoV_NSPer3      TACDCPLTNWEKSIMHDMSANATDDEIALALALFSQEENRLMSDYAGFFELIDDMDNWRL       842
BRNV_Alyth       TKCDVEHTAAEISYVKMIQDDVHPHAYSTNLAISLAQSDRTLWDVKGFRKMVKRIKGYGI       896
CLVA             QIVHKNWTQEERLAIKFALNNATICHYISALNLSCLVEEIDNTLLNEFISLVDMTKLHED       826
DMaV             ALEETPWTTTEIQISETIEAGKNFVDLQGALCLFLLLSESNYAHHKDFMSMVDERHFLET       818
                    .   *  *     .     .          * :     .:         * .::.  .

SMoV_NSPer3      DTPP-RAKKADLQAYVNRTWADYNDRLRFLMCQHFDRTKSARNSYFQRLKTLKDDIRSWS       901
BRNV_Alyth       DDVP-EPVDEQTRNFVRQAWREFSDRDRFLIYRYFEINDTLKQKALAKVATIKEEIKSWS       955
CLVA             GTVTSQNKNEALAEALREFWDKLSPRGRKLLYVIAEYRC---VRYSSFFAGLRSVIRDFN       883
DMaV             GEIKCPGKDAVVERIAQEHWDSLSDAVRYITREIWKKRS--ESEIFAVVTELKNILNSMS       876
                 .       .        .. * . .    * :      .             .  ::. :.. .

SMoV_NSPer3      VVGAWNTLPMGAKWLVGIIALFSFGASLIWLLSKVMAMHTWNPMEMLGVFLGSRSF---I       958
BRNV_Alyth       VAGCWNAMPMGVKWMIGVIALFSFGCVLVDFLHTLMSMRSWSPVDILGNVLGASGAFGVL       1015
CLVA             CAELWARVPMWVKFSLGILALYAGSCVLLRAFDKISTLISLSPTHILATLIGSSSL----       939
DMaV             CVKLWNTMPFWLKWAVGTMALFAGGAVLFKGIRMISSILLVKPAQYLACLLGISSITESE       936
                  .  *  :*:  *: :* :**:: .. *.  :  : ::   .* . *. .:*  .

                  NTB-VPg                       VPg-Pro
                cleavage site                cleavage site
SMoV_NSPer3      EVATEQGGYAESGSNTQAPIYRHKRVRAYEQGASDS-QVMDLNDSEKIEAI---KKAQGI       1014
BRNV_Alyth       AFTSEGGSYAGSGVNIEATKYRDKRIKPYSQGGDGSGANFTYNELESIEAI---KKGQYL       1072
CLVA             AQGTSSGGD------ERVSRAMRRSVRAFSAQGEMSVQVPHQGAWEKCEKARVHLEGFSV       993
DMaV             LQGVSSGGS------ELVGRATRRVVRGFQLQGGAAEDIPHVGAWAKCEQPRVMIDGVFI       990
                     . *.          .      : :: :.  .  :        .    . *       ..  :

                              His of protease catalytic triad
SMoV_NSPer3      LVFSKNDGKSAAAAVTIFKDHQFLITTHELALLNFSKGCILTMRSGASYAIYIDAGNVVV       1074
BRNV_Alyth       LSYSNTCGLSGVAAVFMYKDHSFLISTHEADWINYNKTCYMVGRDGTSREVSLAASGVKK       1132
CLVA             -TTG----RPFAVYGVAYAERKIVCTTHTINELDVMQPIFLR-TQDIAYTCFIRKDAIQY       1047
DMaV             -PELSATGMFGRIYGVMIGNRKILIPSHMVKMLNWSLPCTISNDRDVSARFYLRPEQVTY       1049
                                   ::.::  :*    ::       :    . :    :    :

SMoV_NSPer3      GSK-----GGIKDPISVVKVSTYFGMAKACTGSIMFDFGTYCEGHHNGIVIPNAHKSLDQ       1129
BRNV_Alyth       VTR-----DGFIEPVCSIDILPTTPVGKASSQSVWYNFHKTNEGRKVGVIVPNNRKDMAQ       1187
```

```
CLVA             EQYKNSQGQTILGNLASVKYSNLTPAVPAFT-AFVPNYSKSLPNNENAWIMANLSPLLSA       1106
DMaV             QTTV-ESARVKYGNLAVINYSSGIPAFPHFS-GIDYDRAISGPTSFKGYIMDNLKGLVDY       1107
                               :. :.           : ..  :          . :: *    :

                                             Cys of protease catalytic triad
SMoV_NSPer3      SVLKSTFSRRHEVIDIHNDK-GRVVWQANNLMTVPIYHQVGHCGRLLLARDEAKCLKIVG       1188
BRNV_Alyth       ECTKVHFRRNKEKTDCFNYPGSKGGWSAHDLLHAKAPHGNGMCGRLLLSHGPAGNLLVVG       1247
CLVA             VPLKV-IANKETMVDQFKFDLNNTVWEKRDCTIVKSVGKAGMCGRLLLVE-RHNTLAIAG       1164
DMaV             -PIVT-VFERKSVKDEIELPNTNLSWRKDRILQCNEPGEQGMCGRLALVE-KNGTLLIVG       1164
                       . ...   *  :    .   *               * **** * .     * :.*

                  His of protease                   Pro-Pol
             substrate-binding pocket             cleavage site
SMoV_NSPer3      VHVAGIVIQEKYISLFSEINAIHKSAE---VAVQQGMDIDI-LELVEPETKTEMVVKIGQ       1244
BRNV_Alyth       LHVSGGGLNEKATSIFGGIDGSYKDTEDGKFYQQGDLDVHM-KEFIEPEFITEMVDKIGR       1306
CLVA             MHTYGREYP--PLSGFADIPDFVTNPVI--VAQGPSDDAEFI-YFDAEEEITPMVSKIGY       1219
DMaV             MHNFGNARN----CAFSDIPSEFKPAD--------ELQSEFSMTETPLKQITEMVSQVGF       1212
                 :*  *        . *. *    .              : .:       :  * ** ::*

SMoV_NSPer3      VAHGQQFRPASRTAIIKSQIHDTL---WRAPETEPTVISPTDPRVPYQFDPYTAGIMKFE       1301
BRNV_Alyth       VDPSQQFRPSQGTSIIKSEIHDDL---WRRAETCPTVLTRSDPRPEVPFDPYQAGIRKFV       1363
CLVA             VSTAVP--QLSKSQIEESPIFDSLCQKLGDPKVAPTILSKHDPRPPTPYDPYSAGIRKFD       1277
DMaV             LDAPVP--QLRTTQIEKSPIFDELEDLNGSALTEPTILSVTDPRPPEPFDPYLQGIHKFE       1270
                 :            : * :* *.* *         . **:::  ***    :***  ** **

SMoV_NSPer3      KEVGPLDFTDPDSHESTVVQDITEELAREKKAIGGFALDTVCSDDVAINGVEGVPYAERL       1361
BRNV_Alyth       KEVGPLDFQDEDSTENIVLVDIAEELLSKRREAGGFELDTVLDTHAAINGVDGMEYAEPL       1423
CLVA             KEVGPFDWSEESDLQ-FACAEILDTW--QTKKPEKFAIETVCSLDVAINGVHGLPFAENF       1334
DMaV             KQAGPFDFSEESDLD-LAQKNISSEW--ERFRPEQFDVDTVTTLEVAIQGIDGLDYAESL       1327
                 *:.**:*: : .. :  .  :* .     :      * ::**   ..**:*:.*: :** :

SMoV_NSPer3      VMSTSEGYPFVLSRKAQDTGKFRFFDKD-GDKWIAKDEVLDDLHELEESIKSEDFSGGII       1420
BRNV_Alyth       VMGTSEGYPYVLERQPGDVGKFRYFSKN-LYHWELNEEPKKELDELEESVARDDFDGKII       1482
CLVA             PIGTSEGFPWVLDRARNESGKERFFEEVAPGHRVPRGSWVEDIAEIEEAAYRGDY--LTT       1392
DMaV             PIATSEGFPYILERRAGDTGKERYFEEI-NGKRIPKGDWVQDIDQIERAAVAGNL--EIY       1384
                  :.****:*::*.*   : ** *:*.:     :   . .  .:: ::*.:     :

                           RNA-dependent RNA polymerase motifs
SMoV_NSPer3      TIACAKDEKTKIKKVREVPKTRIFEILPFHYNILVRKYYLFFMQFIMSLHDFLPCKVGLN       1480
BRNV_Alyth       TIACAKDEKTKLVKVYEKPKTRIFEILPYHYNILVRKYYLFFMQFIMKMHNILPCKVGLN       1542
CLVA             TITCAKDEKTKLDKVFVNPKTRIFEMLNFCLNLVIRKYFFFWMQWMMSLHSDLPCKVGIN       1452
DMaV             TMACAKDEKTLKKKIYETPKTRIFEILPFTFNLVIRKYFLFWMQWMMKNHLNLPCKVGLN       1444
                 *::*******   *:   *******:* :  *:::***::*:**::*. *  ******:*

SMoV_NSPer3      VYSKSWDTMHAEHNRFAYHFNGDYTGFDTATPRVLMMRIADMVSELACDGRENAIVRRNL       1540
BRNV_Alyth       PFSQDWDEMHAEHTRFDHHFNGDYSGFDTGTPRQLLLKFADLISDLAGDGRRNKTIRRNL       1602
CLVA             PFSYDWDIMAMKHSAYANHFCGDYSGFDTNTNVELVVRIGDMISDMADDGPRNRVIRRNL       1512
DMaV             VFGFGWDEMKFKHGAYSHHFCGDYSGFDSNTNVAMLDMVADMISDFARDGARNRLIRRNL       1504
                  :. .** *  :*  :  ** ***:***: *   ::  ..*::*::* ** .*  :****

SMoV_NSPer3      MRMAVERRILVLRDLYQVKGGTPSGFALTVIINSVVNQFYLMWAWRKIMSRIDPGLVPYR       1600
BRNV_Alyth       MQLAVDRRILVLAELFHVRGGTPSGFALTVIINSMVNQFYLMWAWRKIMQRISPTMVTYR       1662
CLVA             LRCVLNRQVIVGRDLYKVKGGTPSGFALTVMINSVVNEIYLKMAWFGLSRKYDPLLARDA       1572
DMaV             MHAAVTRRMIVGRNIYKIIGGTPSGFALTVMVNSIINELYLKMAWFNLSKQHFPEISRDA       1564
                 :: .: *:::*  ::::: ***********::**::*::**  **  :  :  * :

SMoV_NSPer3      VMRSHCTFSVYGDDNVVSFSLQVKDMYNLVTIASELKVIGVNLSDGKKTGNLVKWMDFSD       1660
BRNV_Alyth       IMKSHCTFSVYGDDNVVSFSLAVRDMYNLCTIADELKTIGVTLSDGKKTGVLIKWSKFDD       1722
CLVA             DLRHHVCMSVYGDDNVVSFSHNVANWYNLVTISSYLRPFGIKLTDGQKTGVMVPFTSFDK       1632
DMaV             DLSHNVHISTYGDDNVVSFSAQVSPWYNLVTVADYLKRYGVKLTDGKKTGNIVPHMPFSE       1624
                  :  :  :*.**********  *   *** *::. *:  *:.*:**:*** ::    *..

SMoV_NSPer3      LDFLKRRWVLSSGQ-GFLCPLDKSAIEERLFWVRTSEDSVETLDDNCYSALMEAFHHGRE       1719
BRNV_Alyth       LDFLKRRWELEPGH-GFKCPLDKMAIEERLFWVRKSEDNMESLDDNCYSALMEAFHHGPE       1781
CLVA             IDFLKRKWVQGD-KGWFRCPLDPVSIESQLYYLKRSDDPIDALRVNVDNALREAFQHGTD       1691
DMaV             IDFLKRRWVLDDHRSWYRCPLDKTSIEGQCYWIKKSESCYEAMKINIENSLREAYQWGEE       1684
                 :*****:*     :  : ****  :** : :::: *:.  :::  *   .:* **:: * :

SMoV_NSPer3      YFQFLRTKIQDAYDKAGLFQPHLLHFNEAQAIWLEQHSVEPINDYLDGIKKEVLPLTAGR       1779
BRNV_Alyth       YFQDLRGKILDGYQSAGLEAPILLHYQEAKTIWFEQHKVGAESDFFAGVQNHLLPSIVGK       1841
CLVA             YFVRIKCAIVSSLQEA-QIDYLPPSELDCARFWAEQRSCDHLPIHFADPRYRAINILDTK       1750
```

```
DMaV              YFTHWRGRLSIALNRKRLYDVYLPTLNDAARFWNDQRENASLPIHFEDPSARIKYIASSK    1744
                  **   :  :  . :             :.  :* :*:.      .: .   .       :

SMoV_NSPer3       DVLRVITPTIDAVSVKGYNRVHRELAYRRTKVFLDPKAKEIMWLRSDQAAHLNIPASLNK    1839
BRNV_Alyth        DVVRTISTTIDWSSIRRYNEAFASGKYTRTKVFLDPLAKEIQWLKSKSGPHLSIPASLNK    1901
CLVA              VVQTDVGFSMS--VHEYRNRREEME--KLNRLCIIGGDSKLETGLTVNGAKFAVVGAKN-    1805
DMaV              EGHYDNVHCLTARSVNRTNQRNLIN--KIDTVWISAGEKQVRYKKTFEGATHAYVPTLTK    1802
                           :     .  *.           : :    .::    : ..       : .

SMoV_NSPer3       -RNFEGIARDVAK---LLRGEKCCIVEGTCGINSFALALAIGFLRKELTSVGCVNLLASY    1895
BRNV_Alyth        -DTFQSIIEGINR---VVPGEICV-VDGRGDKSGLVVALALAMDRAEISQAQGHNMLLAL    1956
CLVA              -SELVDSLARLFKIGFFT-GKK-------------WVFSA---LNPSLAHVVHICLLNVT    1847
DMaV              PEQLAKICKEIYKDKQNTCEKEFCTM-GSDPRNEIAIITD---QDQPLAHAVGMALVLKY    1858
                     :      : :       :               :          :: .    ::

SMoV_NSPer3       ------------SSSDASYYTGLGMLTAVLG-----------------------------    1914
BRNV_Alyth        ------------VVTNKEVEYGLQLFNVLAATSRPAVSLEPKFTTSPTELVKGIVGVEVY    2004
CLVA              KEHRLETVQYVSQLDDKSYKFMIELNAKIGLVPKPLLE--------------E-------    1886
DMaV              HRDFGKKQVWLSGLDDKAYKAFVNVMNFM-------------------------------    1887
                                 :      : :   :

SMoV_NSPer3       ------------------------------------------------------------    1914
BRNV_Alyth        KVATLVKQLPFGSVGPSLKGRMALFYSDGGFDYSHDKYHYTSQGWPAEIADLAERLGGFN    2064
CLVA              ------------------------------------------------------------    1886
DMaV              ------------------------------------------------------------    1887


SMoV_NSPer3       ------------------------------------------------------------    1914
BRNV_Alyth        SCLVQKYDKGAYIPFHSDDEPCYNLDESIVTVNLDGRATFIVRNKTTNKECRQELYHGSI    2124
CLVA              ------------------------------------------------------------    1886
DMaV              ------------------------------------------------------------    1887


SMoV_NSPer3       ---------------------------------------            1914
BRNV_Alyth        IEMLPGCQKLCKHSVVVKDQGRVSLTFRRQRRTMKGAPL            2163
CLVA              ---------------------------------------            1886
DMaV              ---------------------------------------            1887
```
